# Avascular Necrosis of Femoral Head: A Metabolomic, Biophysical, Biochemical, Electron Microscopic and Histopathological Characterization

**DOI:** 10.1038/s41598-017-10817-w

**Published:** 2017-09-06

**Authors:** Aswath Narayanan, Prakash Khanchandani, Roshan M. Borkar, Chandrashekar Reddy Ambati, Arun Roy, Xu Han, Ritesh N Bhoskar, Srinivas Ragampeta, Francis Gannon, Vijaya Mysorekar, Balasubramanyam Karanam, Sai Muthukumar V, Venketesh Sivaramakrishnan

**Affiliations:** 1 Disease Biology Lab, Department of Biosciences, Sri Sathya Sai Institute for Higher Learning, Prasanthi Nilayam, Puttaparthi, Anantapur District, Andhra Pradesh India; 2 Department of Orthopaedics, Sri Sathya Sai Institute of Higher Medical Sciences, Prasanthigram, Puttaparthi, Anantapur District, Andhra Pradesh India; 3 National Centre for Mass Spectrometry, CSIR-Indian Institute of Chemical Technology, Hyderabad, 500 007 India; 4 Raman Research Institute, C.V Raman Avenue, Sadashivanagar, Bengaluru, Karnataka India; 5 Jan and Dan Duncan Neurological Research Institute, Baylor College of Medicine, One Baylor Plaza, Houston, 77030 United States; 6 Department of Pathology and Immunology, Baylor College of Medicine, One Baylor Plaza, Houston, 77030 United States; 7 Michael E. DeBakey Veteran Affairs Medical Center, Houston, Texas United States; 8 Department of Pathology, M. S. Ramaiah Medical College and Hospital, Bengaluru, Karnataka India; 9 Department of Physics, Sri Sathya Sai Institute for Higher Learning, Prasanthi Nilayam, Puttaparthi, Anantapur District, Andhra Pradesh India; 10 Department of Biology and Cancer Research, 1200, W. Montgomery Rd, Tuskegee University, Tuskegee, AL 36088 United States; 11 Advanced Technology Core, Baylor College of Medicine, One Baylor Plaza, Houston-77030 United States

## Abstract

Avascular necrosis of the femur head (AVNFH) is a debilitating disease caused due to the use of alcohol, steroids, following trauma or unclear (idiopathic) etiology, affecting mostly the middle aged population. Clinically AVNFH is associated with impaired blood supply to the femoral head resulting in bone necrosis and collapse. Although Homocysteine (HC) has been implicated in AVNFH, levels of homocysteine and its associated pathway metabolites have not been characterized. We demonstrate elevated levels of homocysteine and concomitantly reduced levels of vitamins B_6_ and B_12_, in plasma of AVNFH patients. AVNFH patients also had elevated blood levels of sodium and creatinine, and reduced levels of random glucose and haemoglobin. Biophysical and ultrastructural analysis of AVNFH bone revealed increased remodelling and reduced bone mineral density portrayed by increased carbonate to phosphate ratio and decreased Phosphate to amide ratio together with disrupted trabeculae, loss of osteocytes, presence of calcified marrow, and elevated expression of osteocalcin in the osteoblasts localized in necrotic regions. Taken together, our studies for the first time characterize the metabolomic, pathophysiological and morphometric changes associated with AVNFH providing insights for development of new markers and therapeutic strategies for this debilitating disorder.

## Introduction

Avascular necrosis of femoral head (AVNFH) is a progressive, multifactorial and challenging clinical problem that is on the rise^1, 2^, mostly affecting the middle aged male population in the most productive age group of 25–50 years. Clinically, AVNFH is a pathological state with multiple etiologies associated with a reduction in the vascular supply to the subchondral bone of the femoral head. This results in osteocyte death and progressive collapse of the articular surface followed by degenerative arthritis of the hip joint. In majority of patients, non-traumatic AVNFH is either associated with use of alcohol^3^, glucocorticoids^4^ presence of hematologic disorders (for example: sickle cell anemia^5^, thalassemia^6^, polycythemia^7^, hemophilia^8^, myeloproliferative disorder^9^, metabolic disorders (Gaucher disease)^10^ as well as conditions such as hypercholesterolemia^11^, pregnancy^12^, chronic renal failure^13^, hyperparathyroidism^14^, Cushing’s disease^15^ etc. In about 30% of patients however, etiology of non-traumatic AVNFH is unclear and hence these are termed Idiopathic^16^. The fact that AVNFH is sometimes seen in twins and in familial clusters suggests that genetic factors may be involved^17^.

Limited insights into the association of AVNFH with coagulation defects as well as lack of suitable animal models^18^ has resulted in limited knowledge on the pathogenesis of AVNFH. This in turn has hampered the development of predictive markers for disease initiation or progression. Currently, non-invasive diagnostic tests to detect AVNFH include plain radiography, magnetic resonance imaging (MRI), computer assisted tomography (CT), skeletal scintigraphy, and single photon emission computed tomography (SPECT). X-ray based radiographic detection are not sensitive enough to detect AVNFH at its onset (stages 0 and 1) while its overall sensitivity for early stage AVNFH is only about 41%^19^.

Various causal factors have been described to be associated with the manifestation of this disease. These include genetic (SNP), environmental, alcohol and steroid abuse, infection^20^ as well as intravascular coagulation^21^. Among the genetic factors, the most well characterized one describes mutations in Methylenetetrahydrofolate reductase (MTHFR)^22^, which is seen in certain populations. MTHFR mutations can impact one-carbon metabolism and alter levels of homocysteine (HC). Alternately, HC can also be altered by change in levels of folate or co-factors like vitamins B12 and B6^23^. Elevated levels of HC has been shown to adversely affect bone strength^24^, while increased plasma concentration of the metabolite are correlated with increased risk for fractures^25^, Furthermore higher levels of HC and its associated metabolite S-adenosylhomocysteine (SAH) have been reported to cause structural alterations in the bone characterized by increased trabecular separation as well as reduced trabecular thickness, number and area^26^.

Intriguingly enough, in the context of AVNFH, studies looking at alterations in HC metabolism as well as delineating ultrastructural changes in the affected bone are limited. To fill in this knowledge gap, we have used a combination of clinical data mining, mass spectrometry, histopathology, immunohistochemistry and biophysical techniques like Micro-Raman spectroscopy, Raman Mapping, Computer assisted tomography scanning analysis and Scanning Electron Microscopy (SEM) to delineate key changes associated with AVNFH in patients.

## Results

**Figure 1:**
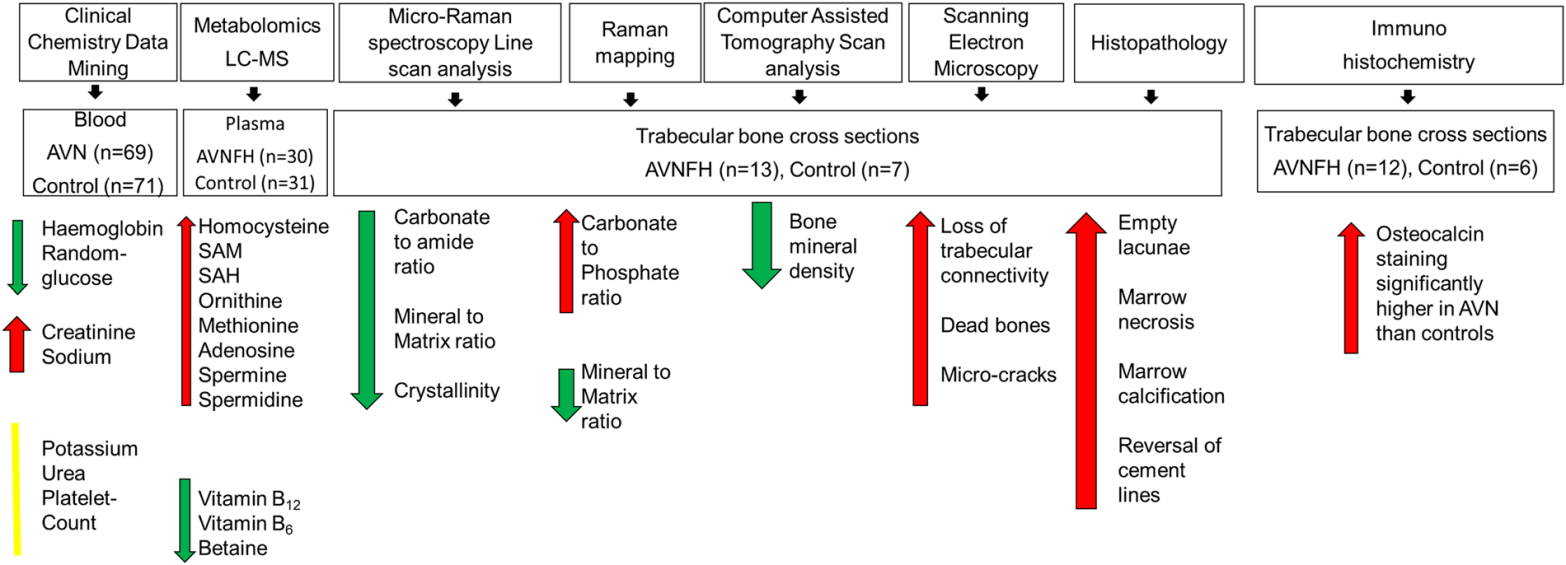
Overview of the comprehensive analysis of AVNFH and control samples. Bone and plasma samples as well as retrospective clinical chemistry data from patients with Avascular Necrosis of Femoral Head (AVNFH) and healthy controls were used in this study. Control individuals visited the hospital for treatment of bone fractures and had no history of AVNFH. Retrospective clinical chemistry data from 69 AVNFH and 71 controls were analysed. In addition, age and gender matched plasma samples collected prior to surgery (AVNFH n = 30) and during routine clinical visit (for controls, n = 31) were used for mass spectrometry-based analysis of selected set of metabolites associated with homocysteine pathway. Bone samples were collected post-surgery (AVNFH, n = 13) or during treatment of fractured bones (controls, n = 7) and used for morphological, histopathological, and biophysical studies. Biophysical studies involved analysis of matrix composition using Micro-Raman spectroscopy and Raman mapping, analysis of bone architecture using Scanning Electron Microscopy (SEM), and analysis of bone mineral density using Computer Assisted Tomography scans as described in the text. Red, Green and yellow arrows/lines indicate elevated, reduced and unchanged levels respectively of assayed parameters in AVNFH versus control samples.

Figure 1 shows the overall workflow of the study. Here we used a combination of clinical data mining, metabolomics, biophysical methods and immunohistochemistry to get deeper insights into biochemical, pathophysiological and morphometric attributes associated with Avascular Necrosis of the Femoral Head (AVNFH). The clinical data mining involved statistical analysis of 7 clinical chemistry parameters for their association with AVNFH using a patient de-identified database consisting of 69 AVNFH and 71 control individuals. Additional insights into altered metabolism and associated metabolite levels were obtained using mass spectrometry analysis of AVNFH and control plasma samples. In parallel, Micro-Raman spectroscopy and Raman mapping were used to compare the chemical composition of AVNFH and control bone samples, while the ultrastructure was studied using histopathology, immunohistochemistry and scanning electron microscopy. In addition, computer assisted tomography scans were used to compare the overall bone mineral density in AVNFH and control bone samples.

Results of these detailed characterization studies revealed elevated plasma levels of metabolites in the methionine-homocysteine pathway, urea pathway and polyamines in AVNFH patients. Further, bone from AVNFH patients showed unique changes in ratio of matrix components which was reflected in ultrastructural alterations describing the loss of trabecular connectivity, presence of micro-cracks, appearance of creeping substitutions, presence of areas containing dead bones and reduced bone density. Taken together, these findings demonstrate extensive bone remodelling in the diseased bone, which is consistent with increased expression of osteocalcin in these samples.

### Alterations in specific biochemical parameters are observed in AVNFH

**Figure 2:**
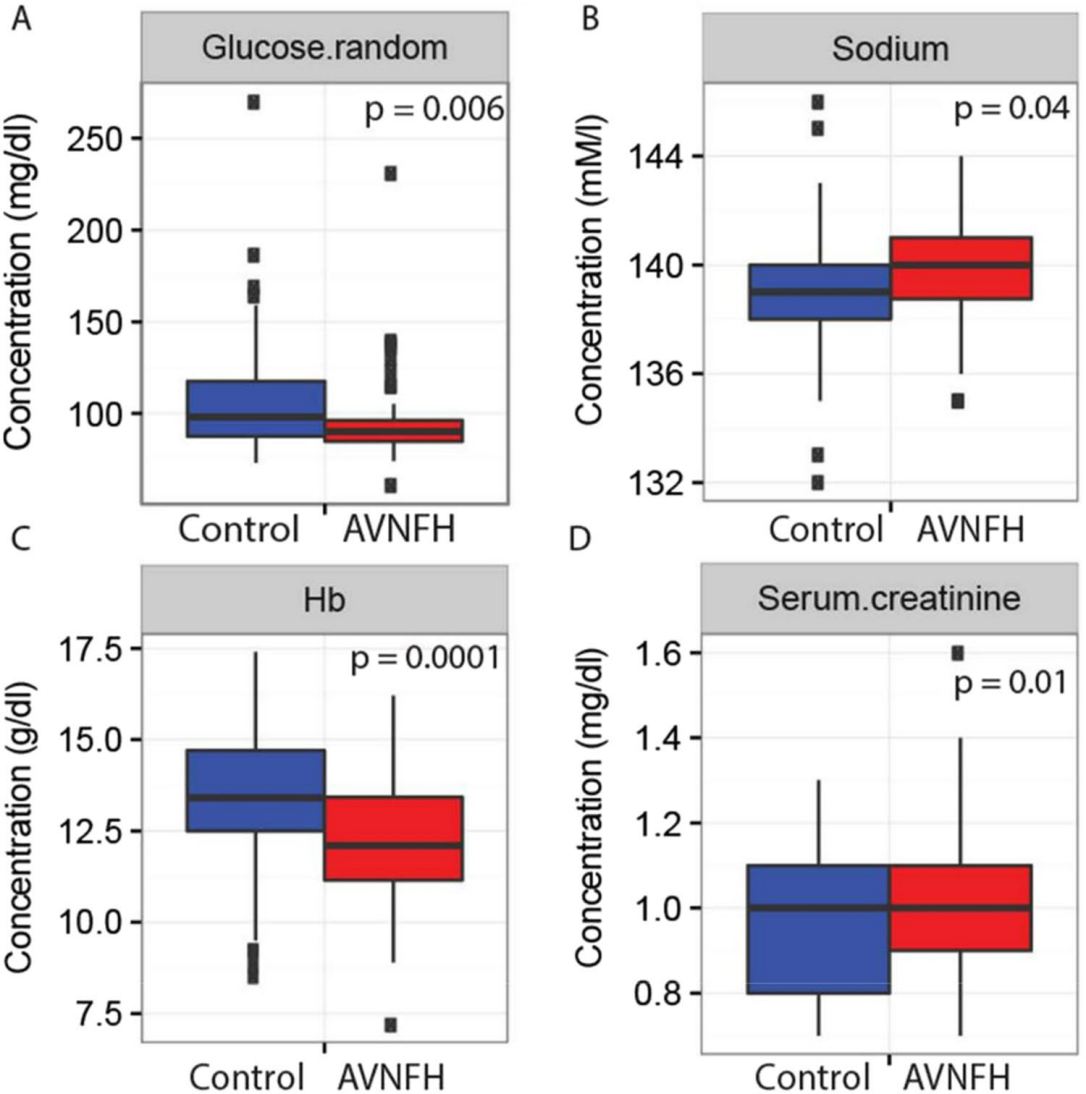
Box plots showing significantly altered levels of clinical analytes in blood samples from AVNFH patients compared to control subjects. X-axis describes the diagnostic groups and Y-axis represents concentrations of the analyte. (**A**) Random glucose, (**B**) Sodium, (**C**) Hemoglobin, and (**D**) Serum creatinine. In all cases p values were calculated using Students T-test. One outlier AVNFH sample having significantly lower values for all the clinical parameters was removed from the analysis.

To begin with, we examined the clinical chemistry values for 7 parameters and compared them between AVNFH patients (n = 68, refer (Supplementary Table S5) for clinical features of the AVNFH cohort) and control individuals (n = 71). Control individuals selected for this study had no prior history of AVNFH and presented at Sri Sathya Sai Institute for Higher Medical Sciences (SSSIHMS) India, for treatment of bone fractures. Supplementary Figure 1 shows the average distribution of the levels of clinical analytes that were compared between AVNFH patients and control individuals. Interestingly, as shown in Fig. 2A and C, levels of random glucose and hemoglobin were significantly down-regulated in AVNFH patients compared to controls. In contrast, levels of serum creatinine and sodium were significantly elevated in AVNFH patients (Fig. 2B,D). These findings indicate presence of an altered metabolism in AVNFH patients, which is consistent with reports describing elevated levels of homocysteine AVNFH patients.

### Targeted plasma metabolomics in AVNFH reveals changes in methionine-homocysteine pathway metabolites

To obtain further insights into altered metabolism and to verify elevated levels of homocysteine in AVNFH, liquid chromatography mass spectrometry (LC-MS) analysis was carried out initially using plasma samples from AVNFH patients (n = 14) and control individuals (n = 14). Specifically, Single Reaction Monitoring (SRM)-based targeted analyses were used to measure the relative levels of metabolites associated with methionine-homocysteine/transsulfuration pathway and polyamine metabolism. Metabolites were extracted using a standardized protocol described under the Methods section. A total of 15 metabolites that included methionine, homocysteine, S-adenosyl methionine (SAM), S-adenosyl homocysteine (SAH), adenosine, betaine, cystathionine, ornithine, arginine, proline, spermine, spermidine and putrescene were measured. In addition, levels of vitamins B_6_ and B_12_ that serve as co-factors in the regeneration of methionine from homocysteine were also measured (Refer to Supplementary Table S1 for list of metabolites and their associated SRM transitions).

**Figure 3:**
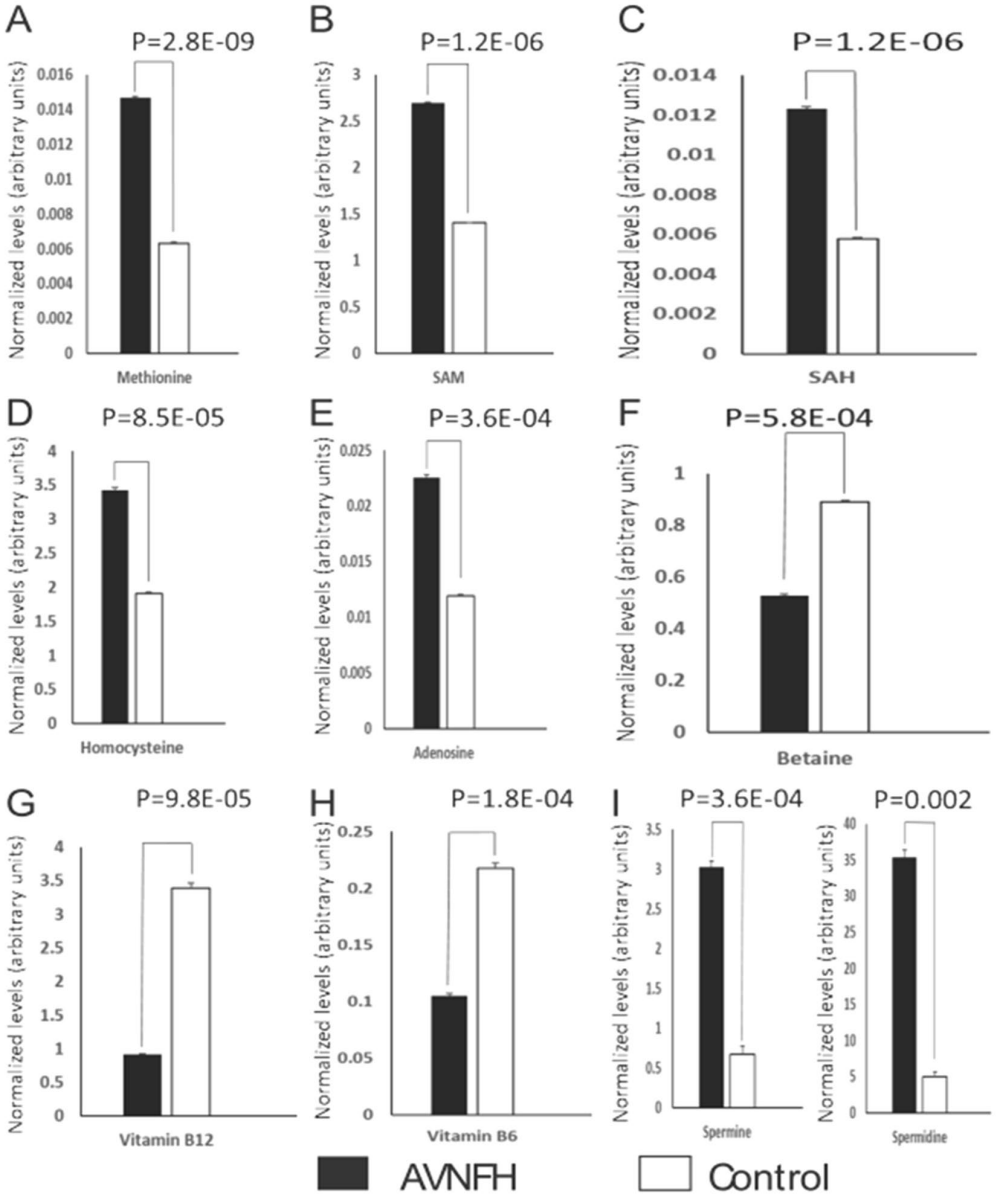
Overview showing normalized levels of selected metabolites in plasma of AVNFH patients (n = 14) and control (n = 14) individuals. Levels of spiked internal standard (see text) were used to normalize the data. P values were computed using Students T-test between the AVNFH (black bars) and control (open bars) groups. Overall levels of metabolites in the folate-methionine Pathway (**A**) Methionine, (**B**) S- adenosyl methionine (SAM), (**C**) S-adenosyl Homocysteine (SAH), (**D**) Homocysteine, and (**E**) Adenosine, were significantly elevated in AVNFH patients. Further levels of (**F**) Betaine, as well as cofactors vitamins (**G**) B_12_ and (**H**) B_6_ were significantly reduced in AVNFH samples compared to controls. Also, levels of polyamines namely (**I**) Spermine and Spermidine were significantly elevated in AVNFH samples compared to controls. P values for each comparison are included in each figure panel.

Importantly, in our initial analysis, levels of methionine, SAM, SAH, homocysteine and adenosine were significantly (P<0.01) elevated in plasma of AVNFH patients compared to age-matched controls (Fig. 3A-E). These findings are consistent with earlier reports that implicate a role for homocysteine in AVNFH^27^. In addition, consistent with elevated SAM levels as well as in line with our earlier observation on elevated levels of circulating creatinine in AVNFH (Fig. 2D), AVNFH plasma had significantly elevated levels of polyamines namely spermine and spermidine (Fig. 3I).

Accumulation of homocysteine in AVNFH patients is further supported by decreased levels of betaine and reduced levels of vitamins B_12_ and B_6_, both of which serve as co-factors in synthesis of methionine from homocysteine (Fig. 3F-H). Taken together, these findings suggest that reconversion of homocysteine to methionine is significantly hampered in AVNFH.

To verify this premise, we carried out a correlation between homocysteine and vitamins B_6_/B_12_ using raw spectral intensity data. Furthermore, we also measured these metabolites in an additional set of plasma samples from AVNFH patients (n = 16) and healthy controls (n = 17), in two independent experiments. Importantly, in each of the three independent experiments, Pearson Correlation analysis revealed a strong negative correlation between homocysteine and vitamins B_6_ (Pearson Correlation range: −0.4 to −0.8, P≤0.05); and between homocysteine and vitamin B_12_ (Pearson Correlation range: −0.5 to −0.8, P<0.05, Supplementary Table S3). In addition, a similar analysis revealed a strong positive correlation between levels of homocysteine and polyamines that included both spermine (Pearson Correlation range: 0.4 to 0.6, P<0.05) and spermidine (Pearson Correlation range: 0.4 to 0.7, P<0.05).

### Micro-Raman spectroscopic analysis of AVNFH Bone shows significant changes in mineral-matrix and chemical composition

**Figure 4:**
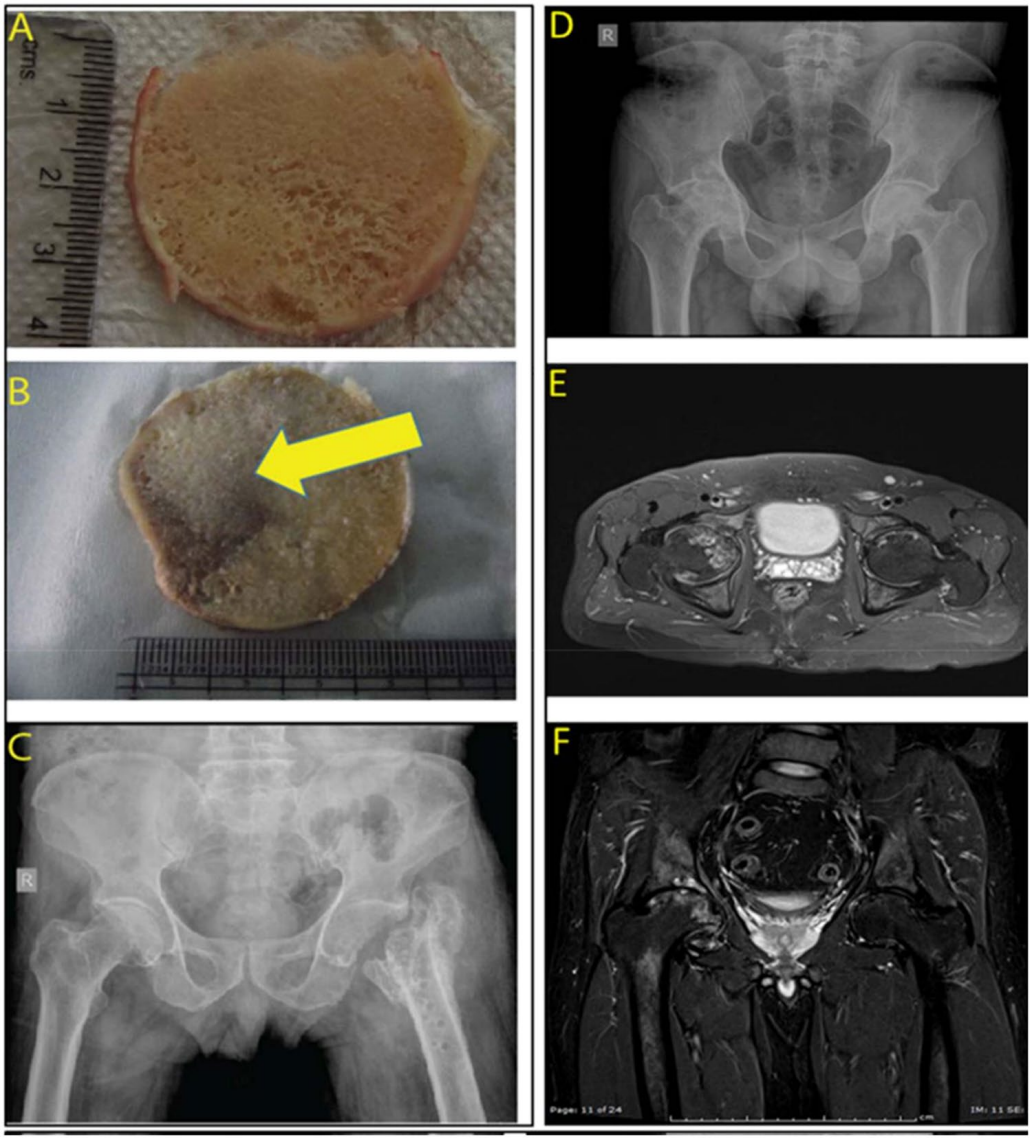
(**A**) Overview of representative AVNFH and control bone samples used in the study. (**A**) A representative gross image of a trabecular cross section of control bone. (**B**) Same as in A, but for AVNFH bone. Region affected by AVN is shown by the yellow arrow. (**C**) X ray image of the pelvic region from a control individual with fractured femur (**D**) X ray image of the pelvic region of a patient with Avascular Necrosis of the Femoral Head (AVNFH). (**E**,**F**) Magnetic Resonance Image (MRI of the pelvic region of a patient with AVNFH.

Having observed significant changes in the biochemical and metabolic profile in blood and plasma of AVNFH patients, we then asked if any of these alterations could be associated with changes in the bone architecture and composition. Figure 4A and B, and Supplementary Figure 2, shows representative cross sections of surgically resected trabecular bones from control individuals (having fractured femur) and AVNFH patients. AVNFH diagnosis was confirmed using X-ray images of the pelvic region (Fig. 4C,D and Supplementary Figure 3) and/or Magnetic Resonance Imaging (MRI, Fig. 4E,F and Supplementary Figure 4). X-ray images of AVNFH patients revealed distinct collapse of the subchondral bone with associated sclerotic changes (Fig. 4C,D and Supplementary Figure 3). Consistent with this, Magnetic Resonance Imaging (MRI) of AVNFH patients shows regions of avascular necrosis in the trabeculae of the femoral bone (Fig. 4E,F and Supplementary Figure 4).

To obtain insights into the chemical composition of the bone, we used various biophysical methods to examine the trabecular cross sections (4–5 cm in diameter, refer Supplementary Figure 2) obtained from 6 AVNFH patients and 3 control individuals. When examined under a stereo microscope, the trabecular cross section of the control bone was found to be homogenous without any gross surface alterations. In contrast, cross sections of AVNFH bones contained both the necrotic avascular region and adjacent non-diseased areas. For all the AVNFH bone samples except one **(**Supplementary Figure 2F), necrotic regions were grossly delineated from adjacent non-disease areas by the clinician post-resection (Fig. 4B, Yellow arrows).

Initially, we carried out Micro-Raman spectroscopy analysis of these trabecular sections. To minimize artefacts in Raman spectral analysis, bone samples were examined without prior chemical treatment. Supplementary Figure 5A shows a representative Raman spectrum for a healthy bone with sequentially labelled peaks that define distinct chemical components listed in Supplementary Table S2. Thus for example, the most prominent peak labelled peak 11 at 1002 cm^−1^ describes the phenylalanine content of the bone while peaks 960 cm^−1^, 1450 cm^−1^ and 1660 cm^−1^ describe the phosphate, collagen and amide content respectively (Supplementary Figure. 5A). Using this annotation, we analysed the Raman spectra obtained from AVNFH and control bones.

Raman spectra were collected across the cross section of the trabecular bone at multiple pre-identified spots (n~16) that were 2600–3000 microns apart. For example, as shown for a representative AVNFH bone (Supplementary Figure 5B), total of 17 spectra were collected, of which 11 were in non-diseased areas (green spots, S1–S6 and S7–S11) and 6 were within the necrotic region (red spots, S1-S6). Importantly, the Raman spectra obtained for control and AVN samples were comparable to the reference spectra shown in Supplementary Figure 5A with well resolved bands for phosphate (960 cm^−1^ and 430 cm^−1^), carbonate (1070 cm^−1^), collagen (1446 cm^−1^ and 1568 cm^−1^) and phenylalanine (1002 cm^−1^).

To begin with, for each sample, all the peak intensities within each spectra was normalized using the corresponding intensity for the phenylalanine peak^28^. We then compared the intensity of the phosphate, carbonate and amide peaks obtained from the control bones with the corresponding intensities obtained from non-diseased and necrotic areas in the AVNFH bone. Following this, we calculated the mineral to matrix ratio (phosphate to amide ratio), carbonate to phosphate ratio, carbonate to amide ratio and the half width of phosphate peak (at 960 cm^−1^), for samples in each of the three groups.

**Figure 5:**
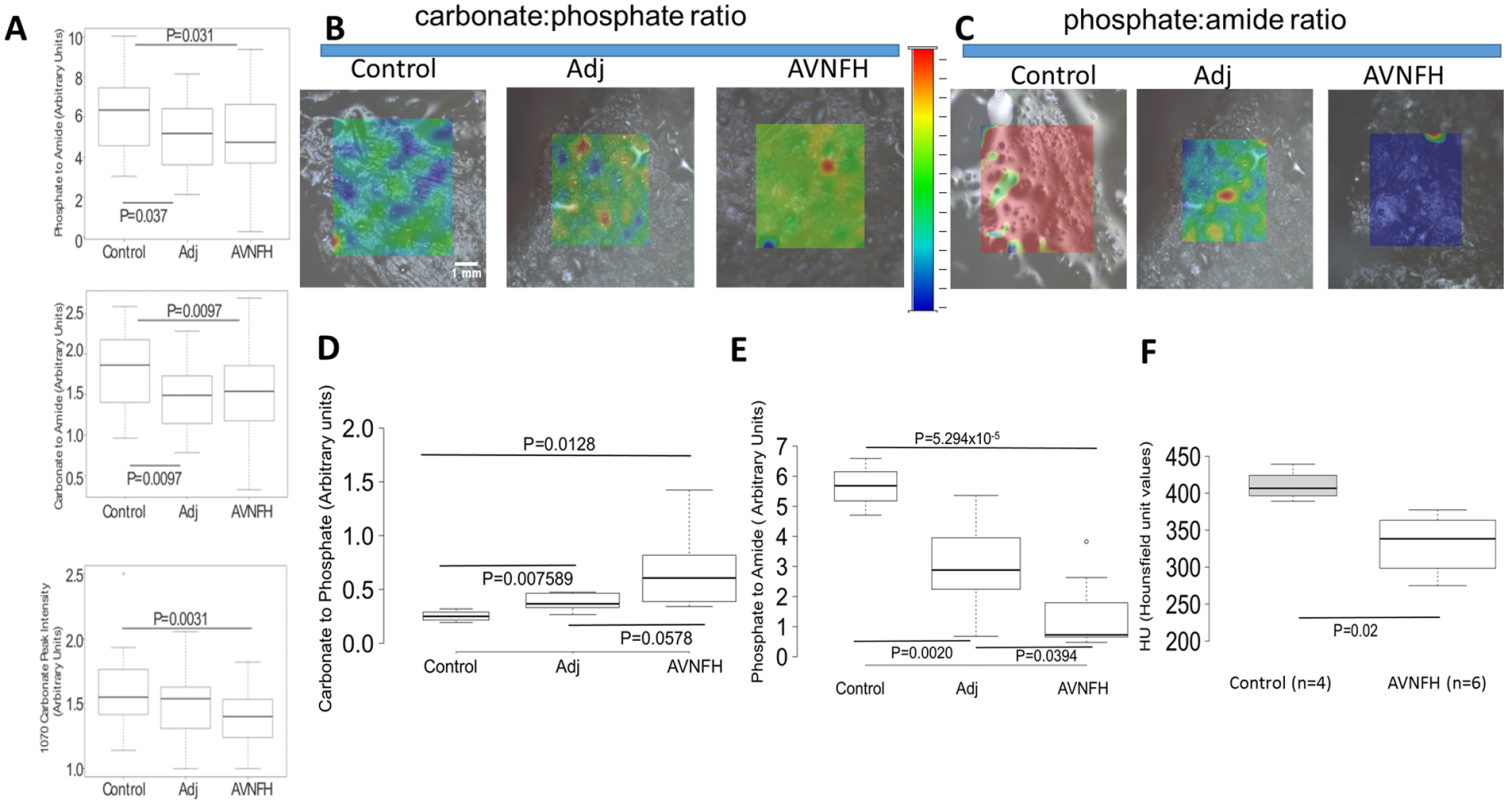
Micro- Raman spectroscopy and computer assisted tomography analysis of AVNFH and control bone. (**A**) Box plot showing the ratio of phosphate to amide peaks (top panel), carbonate to amide peak (middle panel), and carbonate peak intensity, in control, adjacent non-diseased and AVNFH bone samples obtained using micro-Raman line scan analysis. All the three parameters were significantly reduced in necrotic regions compared to control bone. The carbonate peak intensity was not significantly different between control and non-diseased adjacent areas. (**B**) Photomicrographs obtained from Raman mapping analysis showing the distribution of carbonate and phosphate in control, adjacent non-diseased and necrotic regions. Shades of red indicate elevated levels and shades of blue indicate reduced levels. Shades of green indicate intermediate levels (refer to color scale). (**C**) same as in B, but for phosphate and amide peaks, (**D**) box plot showing quantitation of D. Median values derived from one hundred data points is shown. Carbonate to phosphate ratio progressively increased from control to adjacent non-diseased to necrotic areas, (**E**) same as in D, but for phosphate to amide ratio. Phosphate to amide ratio progressively decreased from control to adjacent non-diseased to necrotic areas, (**F**) box plot showing the comparison of median CT intensities quantified by Hounsfield unit values (HU) for Control (n = 4) and AVNFH (n = 6) bone samples. Median HU value that denotes the radio density on a CT scan was significantly lower in AVNFH patients compared to control individuals. Radio density is a surrogate measure for bone mineral density. All P values described in the figure panels were calculated using Student T-tests.

Overall, the control bone had significantly higher values for phosphate to amide ratio and carbonate to amide ratio compared to adjacent non-diseased and necrotic regions (AVNFH, Fig. 5A top and middle panel P<0.05). These ratios were comparable between necrotic and the non-diseased adjacent regions (Fig. 5A). On the other hand, the carbonate to phosphate ratio remained unchanged for all the comparisons (Supplementary Figure 6A). The extent of crystallinity defined by half width of phosphate band (960 cm^−1^) showed a modest reduction in AVNFH regions compared to control (P<0.08, Supplementary Figure 6B). Furthermore, the normalized intensity of only the carbonate peak (1070 cm^−1^) was found to be significantly reduced in necrotic sites compared to control (P<0.01, Fig. 3A, bottom panel).

To further confirm the AVNFH associated changes in chemical composition identified by the Raman line scans, we carried out Raman Mapping studies on an independent group of 6 AVNFH and 4 control bone samples. A total of 100 Raman spectra were individually collected from the adjacent non-diseased and necrotic regions in AVNFH bones as well as from the normal areas in the control bone samples. Interestingly, in contrast to the results of the gross Raman analysis described above, these fine mapping studies revealed significantly higher carbonate: phosphate ratio in AVNFH bone compared to healthy control bone (P = 0.01, Fig. 5B and Supplementary Figure 7). Further, within the AVNFH bone, the carbonate:phosphate ratio showed a progressive increase from adjacent non-diseased region to the AVN region (Fig. 5D and Supplementary Figure 7). In contrast however, the mineral:matrix (959/1660 cm^−1^) ratio was significantly reduced in AVNFH bone compared to control bone (P = 0.0005, Fig. 5C and E and Supplementary Figure 8). Also, within the AVNFH bone, the mineral:matrix ratio showed a progressive decrease from adjacent non-diseased region to the AVN region (Fig. 5E). Taken together, these studies for the first time confirm alterations in the chemical and matrix composition of the AVNFH bone.

### Analysis of computer assisted tomography Scans reveals reduced bone mineral density in AVNFH bone

To determine whether the changes in chemical composition could influence bone mineral density, we obtained radio density of the bone samples that were earlier analysed by Raman mapping. Radio density, used as a surrogate for bone mineral density, was calculated from the computer assisted tomography (CT) scans and represented as standardized Hounsfield Units (HU, Fig. 5F, Supplementary Figures 9–20). Interestingly, 6/6 AVNFH trabecular bone slices that had earlier shown high carbonate:phosphate ratio and low mineral:matrix ratio in the Raman analysis, also had significantly lower median HU values compared to controls (n = 4) (Fig. 5E). 1/6 AVNFH trabecular bone sample was an outlier with high HU values comparable to the control (Supplemental Figure 20). This outlier effect could be potentially attributed to the presence of subchondral sclerotic areas in the AVNFH sample. Taken together, all these findings suggest significant changes in the ultrastructure of the AVNFH bone compare to control bone.

### Scanning Electron Microscopy reveals changes in ultra-structure of AVNFH bone

**Figure 6:**
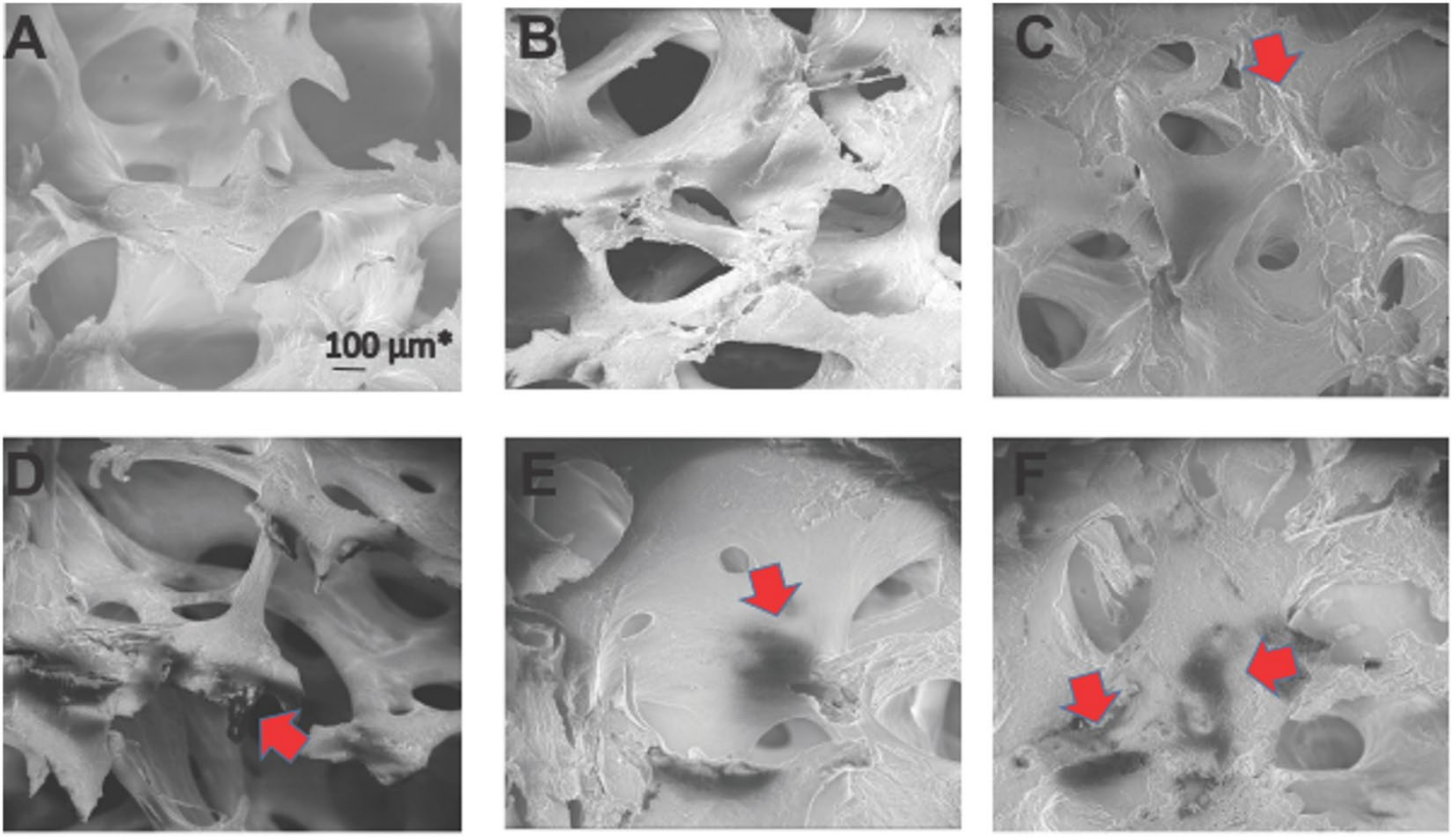
Representative Scanning Electron Microscopic (SEM) images of control and AVNFH bone. (**A**–**C**) SEM images of control bone showing honey comb shaped trabecular arrangement. Regions of bone remodelling is also observed (red arrow). (**D**–**F**) SEM images of AVNFH bone showing perturbed trabecular arrangement. Trabeculae lose connectivity altering the honey comb shape. In addition, regions of dead bones are observed. (Black areas indicated by red arrow). Extensive remodelling of the bone is indicated by red arrows.

In order to study the changes in ultra-structure of AVNFH bone samples, Scanning Electron Microscopy (SEM) was carried out on bone slices, at 30–50 X and 5000x magnifications with minimal pre-treatment of the bone samples. Overall, both control and AVNFH bones exhibited the prototypic architecture (Fig. 6A –F) characterized by the presence of multiple interconnected trabeculae forming honey comb like structures. Further, all the bones displayed resting, resorption and formative surfaces. In the control bone and more prominently in the AVNFH bones, trabeculae were broken with associated loss of connective bridges (Fig. 6A –F).

Interestingly, in the AVNFH bones both the resorption and formative surfaces were predominantly more evident and more extensive compared to controls (Fig. 6D,E and F). Furthermore, AVNFH bones were characterized by the presence of unique dark regions representing areas of bone loss (Fig. 6E,F). These bones also displayed higher surface charging during imaging (data not shown) and calcified marrow regions containing micro-cracks (Fig. 6F). The presence of areas of bone loss (dead bones) was a characteristic feature seen only in AVNFH samples. These findings suggested the existence of extensive remodelling in AVNFH bone compared to the control bone. High power examination of these regions having bone remodelling in both AVNFH and control bones displayed disrupted arrangement of lamellar plates containing disorganized collagen bundles that had lost their polarity (Supplementary Figures 21A,B for AVNFH bone and 21C for control bone).

### Osteocalcin Staining reveals active bone remodelling in AVNFH

**Figure 7:**
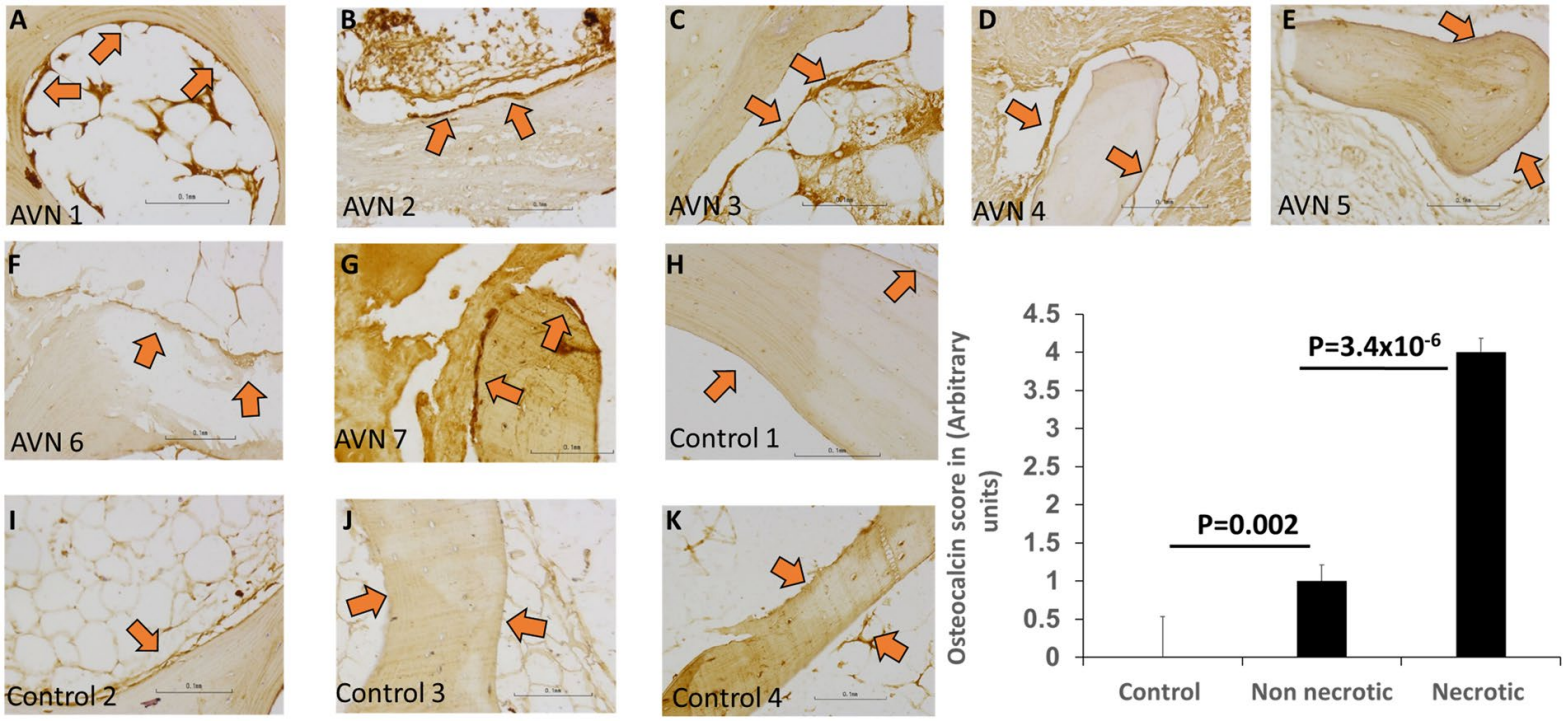
Photomicrographs showing osteocalcin staining in trabecular bone sections from AVNFH patients (panels A–G) and control individuals (panels H-K). Osteocalcin staining in osteoblasts lining (red arrows) the bone were quantified (panel L) by an expert bone pathologist. Overall, strong osteocalcin staining was observed in osteoblasts lining the necrotic areas in AVNFH sample, indicating extensive bone remodelling (panel L). In contrast, osteoblasts lining the control bone showed weak osteocalcin staining (panel L). Interestingly, within the AVNFH osteocalcin (panel L, refer Supplementary Figure 22). In control bones (derived from sites of fracture), osteoblasts proximal to late stage fracture callus showed strong osteocalcin staining, also suggesting extensive bone remodelling (refer Supplementary Figure 22). In control bones (derived from sites of fracture), osteoblasts proximal to late stage fracture callus showed strong osteocalcin staining, also suggesting extensive bone remodelling (refer Supplementary Figure 22). In all cases, non-specific staining was observed in the marrow. All images photographed at 200X (refer to scale in the Inset).

To further verify the presence of extensive remodelling in AVNFH bones compared to control bone samples, we examined the expression of osteocalcin, a marker for bone remodelling. Immunohistochemistry for osteocalcin revealed significantly higher osteoblast staining in AVNFH bone samples compared to control bone samples (Fig. 7A-C, Supplementary Figure 22). Interestingly, within the AVNFH bone, regions distal to the diseased site showed significantly lesser osteoblast staining compared to regions that were proximal to the affected area **(**Fig. 7D and E). Furthermore, osteoblasts associated with early fracture callus in the control bones showed significantly lower staining for osteocalcin (Supplementary Figure 22F and H). In contrast, in one of the control bone samples containing late fracture callus, strong osteocalcin staining was observed in osteoblasts (Supplementary Figure 22G). Unlike its expression in the bone tissue, osteocalcin levels in plasma were not significantly different between the control and AVNFH patients (Supplementary Figure 23). Also, levels of serum parathyroid hormone (PTH), an independent bone resorption marker, were also unchanged between AVNFH patients and control individuals (Supplementary Figure 24). Additionally, levels of CTX (Beta-Crosslaps), an independent bone resorption marker was evaluated in a new independent cohort of 9 AVNFH patients and 9 healthy control individuals. The results are summarized in Supplementary Table S7A and S7B. Normal diagnostic reference was available only for individuals above the age of 30 years, in the clinical laboratory where CTX assays were carried out. Given this, for individuals below the age of 30 years, reference ranges from three independent diagnostic laboratories namely Mayo clinic^29^, Quest Diagnostics^30^ and Arup lab^31^ were used to interpret the data. Importantly, in the age group above 30 years, CTX values exceeded the diagnostic threshold in 3/7 AVN patients (~43%) and only in 1/7 (~14%) controls (Supplementary Table S7A). Along the same lines, for 2 AVN and 3 controls below the age of 30 years, only 1/2 AVN had their CTX values beyond the reference threshold (Supplementary Table S7B). Overall, using this small group of AVN patients and controls, it appears that higher CTX values are more pronounced in AVN patients compared to controls.

### Histopathology of AVNFH bone verifies structural alterations in the trabeculae

**Figure 8:**
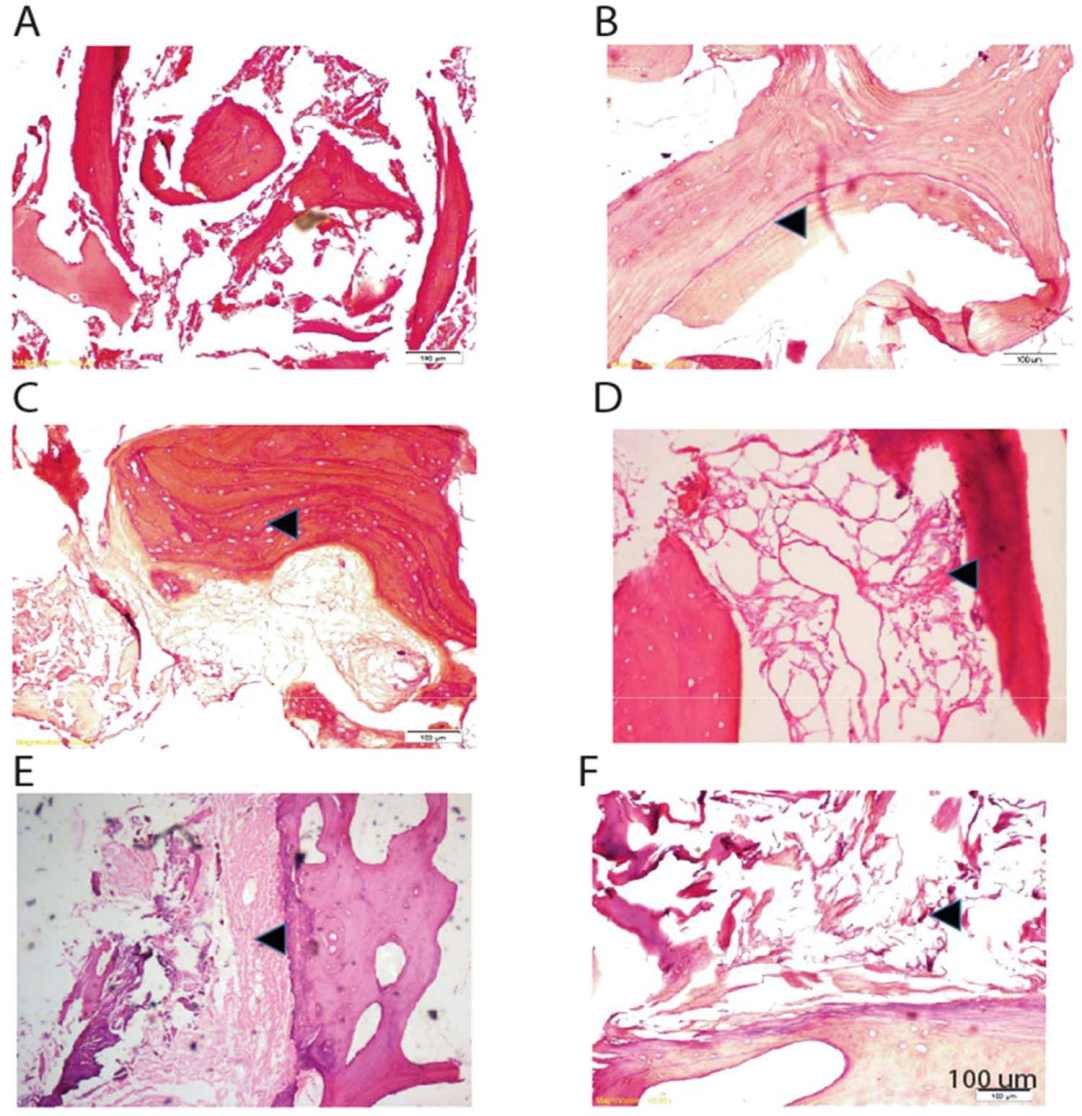
Hematoxylin and Eosin (H&E) - based histopathological characterization of AVNFH and control bone. (**A**) Section of a typical control bone, (**B**) section of a trauma induced AVNFH sample showing a large number of cement lines in the trabeculae reflecting sites of excessive bone-remodelling (black arrow), (**C**) section of non-adjacent diseased region in an AVNFH bone showing large number of empty lacunae in the trabaculae (black arrow), (**D**) section of AVNFH bone showing necrosis of the marrow associated with affected area (black arrow), (**E**) section of AVNFH bone showing the presence of calcified marrow associated with adjacent non-diseased areas of the bone (black arrow), (**F**) section of AVNFH bone showing the presence of poorly vascularized fibrous marrow in the necrotic areas (black arrow).

Histopathology of control bones displayed normal trabecular architecture and marrow devoid of necrosis (Fig. 8A). In contrast, AVNFH bone contained a number of reversal cement lines, both in the necrotic as well as in the adjacent non-diseased region, indicative of extensive remodelling (Fig. 8B-F). This is consistent with the SEM and osteocalcin staining data wherein both formative and resorption areas were predominant in AVNFH. Furthermore, the AVNFH bone sections also reveal a number of empty lacunae resulting from the loss of osteocytes as well as poorly vascularised calcified marrow (Fig. 8E,F).

## Discussion

In this study we have used a multi-pronged approach to characterize AVNFH. A couple of earlier studies have described AVNFH to be a metabolic disorder^32, 33^. These studies describe altered lipid metabolism and altered liver function to correlate with AVNFH^32, 33^ as well as implicated a role for homocysteine in this disease^27^. However, there are no studies to date that examine these metabolic alterations or characterize the levels of homocysteine in AVNFH.

In this study, the initial evidence supporting altered biochemistry in AVNFH was obtained from the retrospective and prospective clinical chemistry data. Interestingly, our analysis revealed significantly lower levels of hemoglobin (Hb) in AVNFH patients. Previous reports have described decreased levels of Hb in AVNFH patients specifically in the context of co-occurrence of sickle cell anaemia^34^. Some of these studies have also reported that lower levels of vitamin B_12_ could result in macrocytic anaemia associated with reduced Hb levels^35^. Interestingly, majority of the AVNFH patients whose samples were analyzed in this study had significantly reduced levels of vitamin B_12_ compared to healthy controls. Although intriguing, additional validation studies are required to confirm the association between vitamin B_12_ levels and reduced Hb in the context of AVNFH. In addition, random glucose levels were also significantly decreased in AVNFH patients compared to healthy controls. This is consistent with data from an independent study that describe significantly reduced levels of glucose in the synovial fluid of pigs wherein osteonecrosis was cryo-surgically induced^36^.

Our findings also suggest significantly higher levels of serum creatinine in AVNFH patients which could potentially be a result of muscle atrophy, a condition reported to be associated with AVNFH in long-term follow up studies in dogs^37^. In addition, an independent study has also described significant association between total plasma homocysteine levels and elevated serum creatinine in middle aged and elderly subjects^38^. Furthermore, in our study, plasma sodium levels were also higher in AVNFH patients compared to controls. Sodium levels in the range of 145–150 mM have been reported to induce secretion of von Willebrand Factor by endothelial cells leading to hypercoagulability and thrombosis^39^. In this context, it is important to note that late stage AVNFH is also categorized as a coagulation disease^40^.

The key finding of this study is the association of elevated levels of homocysteine with AVNFH. In parallel, levels of vitamins B_12_ and B_6_ as well as betaine are reduced. Importantly, this suggests that AVNFH could potentially be associated with altered methionine cycle, in turn resulting in elevated levels of homocysteine. Homocysteine is known to be an inflammatory agent. Multiple studies have demonstrated increased osteoblastic and osteoclastic activity in macrophages and bone cells treated with homocysteine. In line with this, our study further demonstrates existence of extensive bone remodelling in AVNFH. Additional *in vitro* and *in vivo* studies are necessary to unequivocally establish a causal relationship between homocysteine accumulation and bone remodelling in AVNFH. However, if validated, this would introduce the potential of testing inhibitors of methyltransferase or conventional DNMT1 inhibitors for the clinical management of AVNFH. In addition, ratio of homocysteine to betaine or homocysteine to vitamins B_12_ and B_6_, in serum could be evaluated for their potential to serve as non-invasive markers for early detection of AVNFH. In turn, development of such markers for early detection of AVNFH could provide an extended window for treatment thus reducing the number of debilitating hip replacement surgeries.

Betaine is a key substrate for the enzyme betaine homocysteine methyl transferase (BHMT) that converts betaine to methionine. Prior studies^41^ show that high levels of homocysteine in patients and mouse models with hyper-homocystinemia (diet induced or due to cystathione beta synthase deficiency) are associated with reduced levels of betaine. Consistent with this, an independent study demonstrated that betaine supplementation post methionine administration reduced total homocysteine concentration^42^. Taken together, these literature-associated data support our findings describing a reciprocal relationship between the levels of betaine and homocysteine in AVNFH. It will be important to determine whether lowering of betaine levels serves as a metabolic adaptation to maintain higher levels of homocysteine in AVNFH. Preliminary insight to suggest that this might be the case comes from our findings on reduced levels of vitamins B_6_ and B_12_, both of which serve as key co-factors in recycling of homocysteine to methionine. However, our data also demonstrates higher levels of methionine in AVNFH which is counterintuitive in the context of the above argument.

To understand this paradox, it is important to note that some of the earlier studies demonstrated normal to higher levels of methionine in majority of individuals with vitamin B_12_ or folate deficiency^43–45^. For example, serum levels of methionine were found to be normal or higher than normal in 51/60 patients with B_12_ deficiency and in 55/60 individuals with folic acid deficiency^43^. In an independent study^44^, methionine levels were predominantly higher than normal levels in cobalamin-deficient patients when compared to control individuals. Along the same lines, in an independent study^45^ levels of methionine in animals with vitamin B_12_ deficiency was similar to those observed in control animals, despite being fed methionine rich chow. Thus overall, these published literature shows that under conditions of vitamin B_12_ deficiency, methionine levels tend to be maintained within normal levels suggesting alternative mechanisms for methionine repletion such as increased dietary uptake of the metabolite that needs to be verified using additional studies.

All of the above clinical findings highlight the importance of studying altered metabolism in AVNFH. Elevated serum levels of homocysteine are known to be strongly associated with AVNFH^27^, while factors such as alcohol intake, smoking etc., that are known to be associated with onset of the disease, are known to alter circulating levels of vitamin B_12_ an important co-factor in the Methionine cycle. Furthermore, multiple studies have described mutations in MTHFR to be associated with AVNFH^22, 46^. Importantly, our plasma data suggests elevated levels of metabolites in the Methionine cycle leading to the formation of homocysteine while levels of compounds associated with recycling homocysteine back to methionine were significantly reduced. This is consistent with strong and significant negative correlation between levels of homocysteine and Vitamin B_6_/B_12_ in plasma samples. Together these strengthen findings describing elevated levels of homocysteine in plasma of AVNFH patients.

Prior studies have shown that higher levels of homocysteine could promote osteoclast formation^47^ resulting in increased bone loss as well as leading to thrombosis causing atherosclerosis and occlusion of arteries and veins^48^. According to the vascular hypothesis (or regional endothelial bed dysfunction), local microvascular thrombosis could decrease blood flow in the femoral head resulting in onset of AVNFH^49, 50^. Importantly, key metabolic alterations observed in AVNFH samples suggest occurrence of extensive bone remodelling in these samples. Thus for example, elevated levels of polyamines have been shown to be associated with increased osteoblast formation^51^, while elevated levels of SAM and SAH have been shown to induce osteoclast formation by promoting methylation-induced silencing of inhibitors of osteoclastogenesis^52^. Adenosine was also shown to be important for osteoclastogenesis^53^. Furthermore, reduced levels of Vitamin B_12_ have also been associated with osteoclast formation^54^.

Overall, our metabolic analysis highlights important vignettes that promote constant remodelling of bones, a characteristic feature of AVNFH.

AVNFH-associated bone remodelling is further substantiated by our biophysical studies. Micro-Raman spectroscopy is a widely used tool to study the chemical composition of the bone^55^. A healthy bone is composed of hydroxyapatite, carbonate substituted apatite and collagen^56^. Micro-Raman spectroscopy-based line scan analysis of AVNFH bones showed decreased Phosphate to amide I and carbonate to amide I ratio, suggesting reduced crystallinity, a measure of bone strength^57^. In addition, decrease in mineral matrix ratio in AVNFH bones that contain extensive breakdown of trabecular architecture, reveals extensive resorption of the necrotic bone. Furthermore, SEM images indicate a time lag between resorption and formation of new bone in AVNFH samples.

Interestingly, a more in depth Raman mapping analysis revealed an elevation in Carbonate to phosphate ratio in AVNFH areas when compared to controls. This is in line with the results of an earlier study where in necrotic bone of AVNFH was marked by increase in carbonate to phosphate ratio suggesting a trend towards increased resorption^58^. This is further supported by CT scan analysis of samples that were used for Raman mapping that revealed that the radio density (represented as Hounsfield number) associated with AVNFH was significantly lower than the values for the controls. Importantly, as reported by others, the mean number of HU within each region is a surrogate for bone mineral density^59^ and higher Hounsfield values are associated with tissues having higher density^60^. Taken together, these findings indicate extensive structural remodelling in AVNFH bone.

Remodelling in AVNFH bone was further verified using SEM. A hall mark of AVNFH bone when examined using SEM, is the appearance of dead bones and micro-cracks. Micro-cracks are microscopic cracks with length in the range of 30–80 microns^61^. The presence of trabecular micro-cracks in necrotic bone suggest healing defects and has been suggested to result in secondary vascular impairment in the capillaries either by causing compression of non-elastic fat cells or rupture of small intra-trabecular vessels^62^. Defect in bone structure was further visualized using histopathology that revealed absence of osteocytes within the necrotic zones. These could represent areas of dead bone as reported by others^63^. Importantly, necrotic areas containing dead bone appeared dark in SEM images and represented a unique feature seen only in AVNFH samples.

Despite these extensive changes in bone architecture, plasma levels of bone resorption markers like osteocalcin and parathyroid hormone (PTH) did not change in AVNFH patients compared to controls. Our results corroborate with that of earlier reports where in osteocalcin and a set of bone formation and resorption markers did not vary significantly between Osteonecrosis of knee and control patients^64^. Intriguingly however, osteocalcin expression associated with osteoblasts in the sections of trabecular bones were significantly altered in AVNFH compared to controls. Interestingly, osteoblasts lining the bone in areas of necrosis or fracture repair (where extensive bone remodelling is known to occur) strongly stained for osteocalcin. These results are in line with previous reports that show increased osteocalcin deposition in AVNFH areas^65^. In contrast, osteoblasts in healthy bone or in regions distal from sites of necrosis or bone repair showed weak osteocalcin expression. Furthermore, CTX levels, an independent bone resorption marker showed a higher likelihood of being elevated in AVN compared to controls in a small pilot study that needs to be verified in larger patient and control cohorts. The relatively lower percentage of CTX positive AVN in our pilot study is consistent with a case report that describes absence of elevated CTX values in AVNFH that developed secondary to fibrous dysplasia^66^. Importantly, these findings suggest that AVNFH is more likely a localized disease with active remodelling of the bone occurring in specific areas associated with necrosis. Our immunohistochemistry data also showed significant non-specific intense staining in the marrow potentially due to high levels of peroxidase activity in these tissues. Yet another explanation for unchanged levels of bone resorption markers in the small group of AVNFH plasma that we analysed could be that these patients presented with advanced stage disease (stage 3 and 4).

## Conclusions

Elevated levels of homocysteine and metabolites belonging to the homocysteine and polyamine pathway in the plasma are associated with Avascular Necrosis of the Femoral Head. The increase in homocysteine is correlated with a decrease in vitamin B_12_, B_6_ and betaine. Micro-Raman spectroscopy study shows that the bone from AVNFH patients displays decreased phosphate to amide and carbonate to amide ratio indicative of reduced mineralization. Raman mapping interestingly revealed a significantly higher carbonate to phosphate ratio, indicative of bone resorption, as well as decreased mineral to matrix ratio indicative of decreased mineralization in AVNFH bones consistent with the CT scan data demonstrating reduced bone mineral density. Consistent with all of this, SEM and histopathology reveal the presence of disrupted trabeculae and micro-cracks, co-existing with regions of dead bone as well as loss of osteocytes with associated necrosis of the marrow. Taken together, these data suggest extensive remodelling in AVNFH bones which is supported by increased expression of osteocalcin in osteoblasts associated with necrotic sites.

## Methods

### Clinical Samples

Retrospective and prospective data on clinical parameters from AVNFH and controls were abstracted from the SSSIHMS database by a honest broker per approval of the SSSIHMS institutional bioethics commission (Approval number: SSSIHL/IEC/PSN/BS/2012/05) and provided for the study in a de-identified manner by a honest broker. Informed consent was obtained from all subjects. All samples used in this study were collected in a de-identified manner and the methods were carried out in “accordance” with the approved guidelines and regulations. Sections of trabecular bone or plasma from de-identified AVNFH patients were obtained from Sri Sathya Sai Institute of Higher Medical Sciences with approval from the institutional bioethics commission and patient consent. AVNFH trabecular bone sections (n = 13 total) were obtained post-hip arthroplasty. AVNFH plasma (n = 30 total) was obtained from patients visiting SSSIHMS for AVNFH treatment. De-identified control bone (n = 7 total) and plasma (n = 31 total) was obtained from fracture cases with no history of AVNFH. Paraffin embedded tissue sections for histopathology and immunohistochemistry from AVNFH and control samples were obtained in a de-identified manner from SSSIHMS and Baylor College of Medicine.

### Analysis of Clinical Chemistry Data

Clinical data from 69 AVNFH patients (refer Table 1 for summary of clinical parameters) were used for the analysis. This included retrospective data from 55 patients and prospectively collected data from 14 patients currently consulting at the hospital. 1/69 patient was an outlier for all the clinical parameters and hence was excluded from all the downstream analysis. Clinical data from a random set of age and gender matched control individuals (n = 71) was also obtained in a de-identified manner.

**Table 1:** Information regarding the reference ranges of 9 clinical parameters in the plasma of AVNFH and Control patients.

Clinical Parameter	Normal Diagnostic Range	AVNFH (n = 69)	Control (71)
Sodium (mM/L)	135–145	140 (117–144)	139 (132–146)
Potassium (mM/L)	3.5–5.1	4.3 (3.5–5.3)	4.2 (3.3–5.3)
Calcium (mM/L)	8.5–11	9.3 (8.1–10)	9.1 (7.8–10.5)
Urea (mg/dL)	15–45	20 (11–52)	20 (9–42)
ESR (mm/hr)	9–20	17 (2–125)	17 (3–75)
Random Glucose (mg/dL)	70	90 (61–231)	98.5 (78–270)
Hemoglobin (g/dL)	13–18 (men) 11.5–16.5 (women)	12.15 (7.2–16.2)	13.4 (8.5–17.4)
Platelet count (lakhs/cmm)	1.5–4	2.38 (1.11–4.57)	2.3 (1.1–5)
Serum Creatinine (mg/dL)	0.7–1.2 (men) 0.5–1 (women)	1 (0.7–1.6)	1 (0.7–1.3)

Out of a total of 23 clinical measurements that were obtained for each subject, only 9 parameters had values in at least 70% of the total subjects. For these parameters, missing values in patients or controls were computed using the median value for the entries in the corresponding experimental group (i.e., AVNFH or control). The levels for each of the parameter were then visualized relative to the corresponding clinical reference range. To determine the significance in the altered levels of parameters in AVNFH relative to control, a student t-test was applied. Box plots were used to visualize significantly altered parameters in AVNFH and control groups after removing one of the AVNFH outlier samples as described above. Following this, multivariate analysis was performed using PLS-DA on all the variables. Logistic regression model was built using all the variables including age and gender. This analysis used the Generalized Linear Model (glm) function in the Stats package in R. The predictive power for these three parameters was verified using a 10-fold cross validation repeated 1000 times.

### Metabolite Analysis of Plasma samples using Mass Spectrometry

Plasma was extracted from blood samples collected in EDTA coated tubes following standard protocols. For extraction of metabolome, plasma samples were homogenized in 1:4 ice cold water: methanol mixture containing equimolar mixture of 2 standard compounds, Zeatine, ^[15N]^2-Tryptophan. Extracts were de-proteinated by passing through a 3 KDa filter, filtrate was dried, resuspended in injection solvent (water: acetonitrile, 95:5, containing 0.1% formic acid) and analyzed by liquid chromatography-coupled to mass spectrometry (LC-MS/MS). The LC-MS/MS analysis was carried out on a Micromass Quattro Micro™, Waters Inc., Manchester, UK and Agilent 6420 triple quadrupole, Agilent Technologies, Santa Clara, CA coupled to Waters and Agilent HPLC system, respectively. Data acquisition was performed using MassLynx software (Waters) and Mass Hunter workstation software (Agilent). A detailed information regarding the operational parameters of HPLC and LC-MS/MS analysis for the samples is given in the Supplementary methods section.

### Micro-Raman spectroscopy

Micro-Raman measurements were carried out using a Raman microscope (T64000-HORIBA Jobin Yvon, Kyoto, Japan) at Raman research Institute, Bengaluru, India. A Helium-Neon (He-Ne laser excitation wavelength of 633 nm) was used to minimize background fluorescence from the biological specimen. The excitation laser power incident on the sample was fixed at about 2 mW. Each spectrum was recorded using 1800 grooves/mm grating with 80 seconds of exposure time over the region of interest. Multiple Raman spectra were obtained along the diameter of the bone slice at 2600–3000 micron intervals, by focusing the laser beam through a 50XLWD (long working distance) microscope objective. For AVNFH specimens, the spectral acquisition spanned the entire diameter of the bone encompassing adjacent non-diseased areas and AVNFH containing regions. For control specimen, an equivalent number of Raman spectra were obtained along the diameter of the bone slice. Raman spectra were recorded between the wavenumber ranges of 400–1800 cm^−1^ to enable examination of both the mineral and organic phases of the bone tissue. For each of the bone slices, a line scan typically comprising of 10–12 spots was carried out along the entire cross section of the bone. All the data were normalized with respect to the peak intensity of phenyl alanine at 1002 cm^−1^. The normalized data was used for downstream statistical analysis to compare and quantify the mineral-matrix components in the AVNFH bone versus the control bone like bone crystallinity, Carbonate to amide ratio and Phosphate to amide ratios.

### Raman mapping

Raman mapping was carried out using Thermo Scientific DXR Raman microscope emitting 780 nm infrared wavelength at Sri Sathya Sai Institute of Higher Learning, Puttaparthi, India. Laser beam was focused on the sample through 10X objective. Each spectrum was recorded using 400 grooves/mm grating with 10 seconds of exposure time with an estimated resolution between 4.7 and 8.7 cm^−1^ over the region of interest. The excitation laser power incident on the sample was fixed at about 4 mW. The typical spot size impinging the sample was 3.1 µm. The mapping was carried out on two morphologically distinct areas of AVNFH bone sample representing the necrotic and the non-diseased adjacent areas as determined by the clinician, using rectangular grids of 300 × 300 µm^2^. A similar area was also chosen in the control bone samples for analysis. Raman spectra were recorded in an extended spectral range between 100 and 3300 cm^−1^. Raman maps showing the intensities of phosphate, amide and carbonate functional moieties along with ratios for Carbonate to phosphate and mineral to matrix ratio were computed. Median values with associated standard deviations were used to compare between the groups. Two-sided t-test was used to determine the significance of the results.

### Scanning Electron Microscopy (SEM)

The bone specimens stored in PBS at −20 °C were thawed and subjected to a pre-treatment process to preserve the integrity of collagen and remove any cellular and surface organic material, following protocol published by^67^. Notably, for the SEM analysis, the bone samples were not sputter coated so as to avoid any artifacts. Processed specimens were examined using a Carl Zeiss Ultra plus Gemini Electron Microscope (Oberkochen, Germany) at the Raman Research Institute, Bengaluru, India.

### Computer Assisted tomography analysis of Hounsfield units (HU)

CT scans were acquired on multiple trabecular bone cross sections of AVNFH patients (n = 7) and healthy controls (n = 4), using GE Discovery CT 750 HD GST FREEDom edition CT scanner at the Sri Sathya Sai Institute of Higher Medical Sciences Prasanthigram, India. Parameter used to acquire the CT image included: Tube voltage of the CT-scanner: 140 kV, current: 280 mA, slice thickness: 0.625 mm and interval between slices: 0.625 mm. CT acquisition was carried out in high resolution mode using the application software 13MW24.7 V40-PS HD64-G-GTL. CT images were saved in DICOM format using the Synapse software version 4.3.2 Fuji film medical system. U.S.A. Inc. Multiple elliptical Regions of Interest (ROI), each having an area of 50 mm^2^ and perimeter size of 25 mm were superimposed on the reconstructed images of each of the bone slice using the synapse platform. CT intensities were computed in Hounsfield units (HU) for each of the ROIs. Median value of CT intensities was computed across all the bone slices independently for each patient/control sample.

### Bone Histopathology and immunostaining for osteocalcin

Necrotic and normal adjacent areas from the bone slices obtained from AVNFH patients were grossly excised and fixed independently along with the control bone slices in 37% formaldehyde. Following this, all the specimens were decalcified using 10% EDTA, dehydrated, processed conventionally and embedded in paraffin wax. The embedded bone specimens were cut into 5 µm sections using a Leica microtome (Buffalo Grove, IL at M.S Ramaiah Medical College, Bengaluru, India. The sections were stained using Hematoxylin and Eosin and reviewed by two board certified bone pathologists from M.S Ramaiah Medical College, Bengaluru and BCM. These sections along with additional ones from BCM (5 AVNFH and 2 controls) were used for immunostaining using osteocalcin antibody. Prior to osteocalcin staining, tissues were de-paraffinized in xylene and rehydrated in graded alcohols. Endogenous peroxidase activity was quenched with 3% hydrogen peroxide. Slides were blocked by 3% goat serum at room temperature for 1 hour in humidity chambers and incubated first with anti-rabbit *Osteocalcin* (Bioss Antibiodies Inc. Woburn, MA, USA) for 2 h and then with HRP conjugated goat anti-rabbit secondary antibody (Jackson Immunoresearch Laboratories Inc, West Grove, PA) for 40 minutes. The antigen-antibody reaction was visualized after diaminobenzidine (Sigma-Aldrich, MO) and the slides were counterstained with hematoxylin (Sigma-Aldrich, MO) and mounted with Permount media. Positive controls were stained in parallel; negative controls were generated by omitting the primary antibody.

**Parathyroid Hormone (PTH) and Osteocalcin measurement in sera** were carried out using the kits PTH 11972103160 and Osteocalcin 12149133160 (Roche diagnostics), following manufacturer’s protocols at Thyrocare Mumbai and Metropolis Mumbai respectively.

**CTX (Beta-CrossLaps) measurement in plasma** was carried out using the kit 11972308160 (Roche diagnostics), following manufacturer’s protocols at Metropolis Mumbai.

**Experiments on humans and the use of human tissue samples**. We confirm that all experiments were performed in accordance with relevant guidelines and regulations.

## Electronic supplementary material

Supplementary Information
