# Supplementary material for: Avascular Necrosis of Femoral Head: A Metabolomic, Biophysical, Biochemical, Electron Microscopic and Histopathological Characterization
Source: Sci Rep. 2017 Sep 6;7:10721. doi: 10.1038/s41598-017-10817-w (PMC5587540; doi:10.1038/s41598-017-10817-w)
Supplement: Supplementary file 1 — Supplementary Information [file 41598_2017_10817_MOESM1_ESM.pdf]

Avascular Necrosis of Femoral Head: A Metabolomic, Biophysical, Biochemical, Electron Microscopic and Histopathological Characterization.

Aswath Narayanan[1], Prakash Khanchandani[2*], Roshan M. Borkar[3], Chandrashekar Reddy Ambati[9], Arun Roy[4], Xu Han[5], Ritesh N Bhoskar[2], Srinivas Ragampeta[3], Francis Gannon[6], Vijaya Mysorekar[7], Balasubramanyam Karanam[10], Sai Muthukumar V[8], Venketesh Sivaramakrishnan[1*]

Contents

1. Supplementary Figures 1-24
2. Supplementary Tables 1-7
3. Supplementary Methods

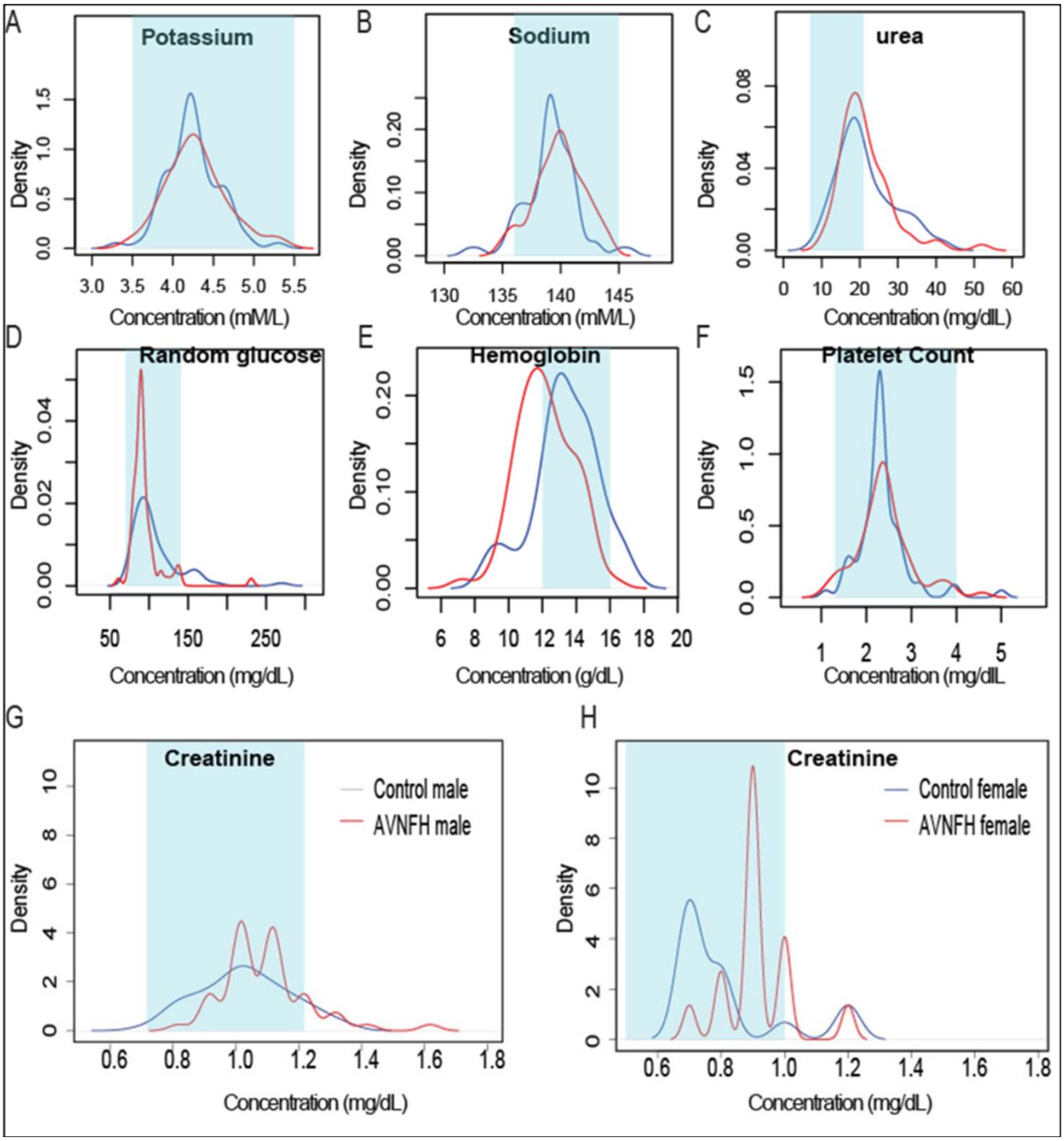

Supplementary Figure 1: Summary of clinical chemistry data in retrospective cohort of AVNFH patients compared to control individuals. For panels A-F, red and blue lines describe the distribution of absolute levels of various analytes tested in AVNFH and control individuals respectively. Area shaded in blue indicates the normal range. X-axis and Y-axis indicate the absolute concentration and estimated density respectively. A) Potassium B) Sodium C) Urea D) Random glucose E) Hemoglobin F) Platelet Count and G, H) Serum creatinine, panel G represents distribution in males (n=56 control and n=53 AVNFH) and panel H represents distribution in females (n=15 each control and AVNFH).

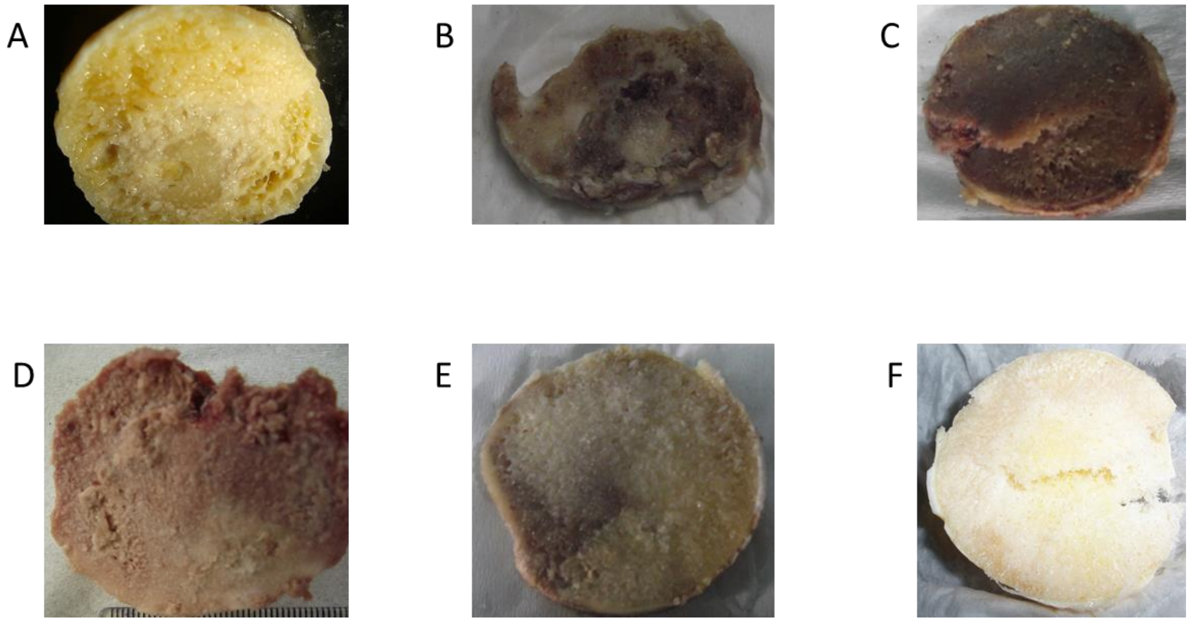

Supplementary Figure 2: Gross images of trabecular femoral head cross sections of A) AVN patient 1, B) AVN patient 2 C) AVN patient 3, D) AVN patient 4, E) AVN patient 5 and F) AVN patient 6

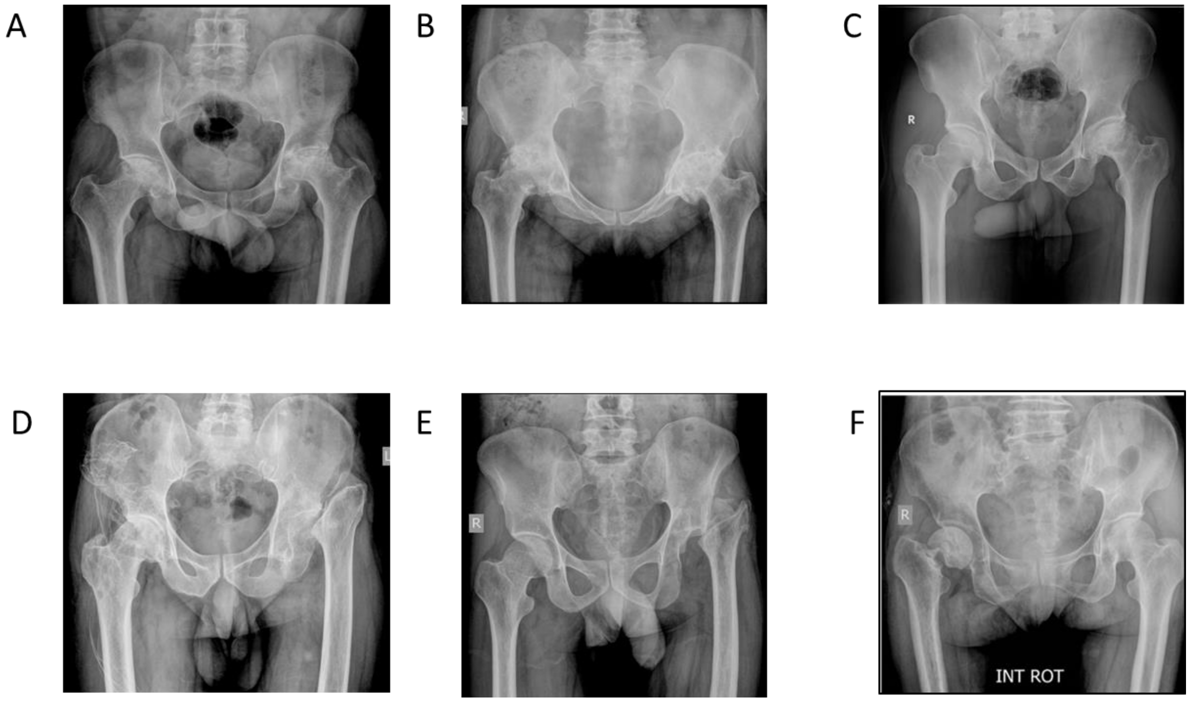

Supplementary Figure 3: A) X ray of AVN patient 1, B) X ray of AVN patient 2 C) X ray of AVN patient 3, D) X ray of AVN patient 4, E) X ray of AVN patient 5 and F) X ray of AVN patient 6

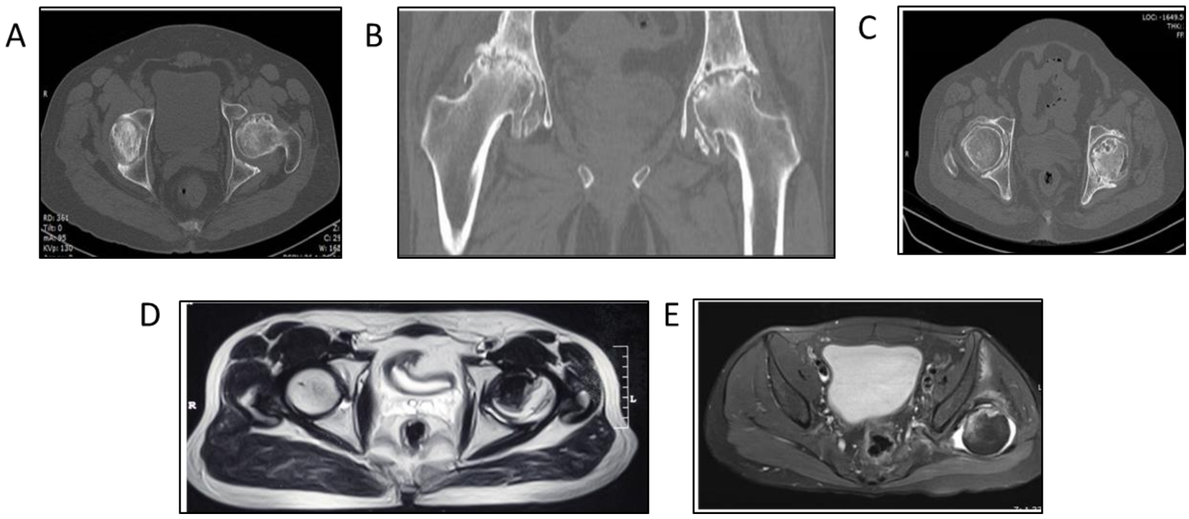

Supplementary Figure 4: A) CT scan image of AVN patient 1, B and C) CT scan images of AVN patient 3 D) MRI Image of AVN patient 2, E) MRI image of AVN patient 5

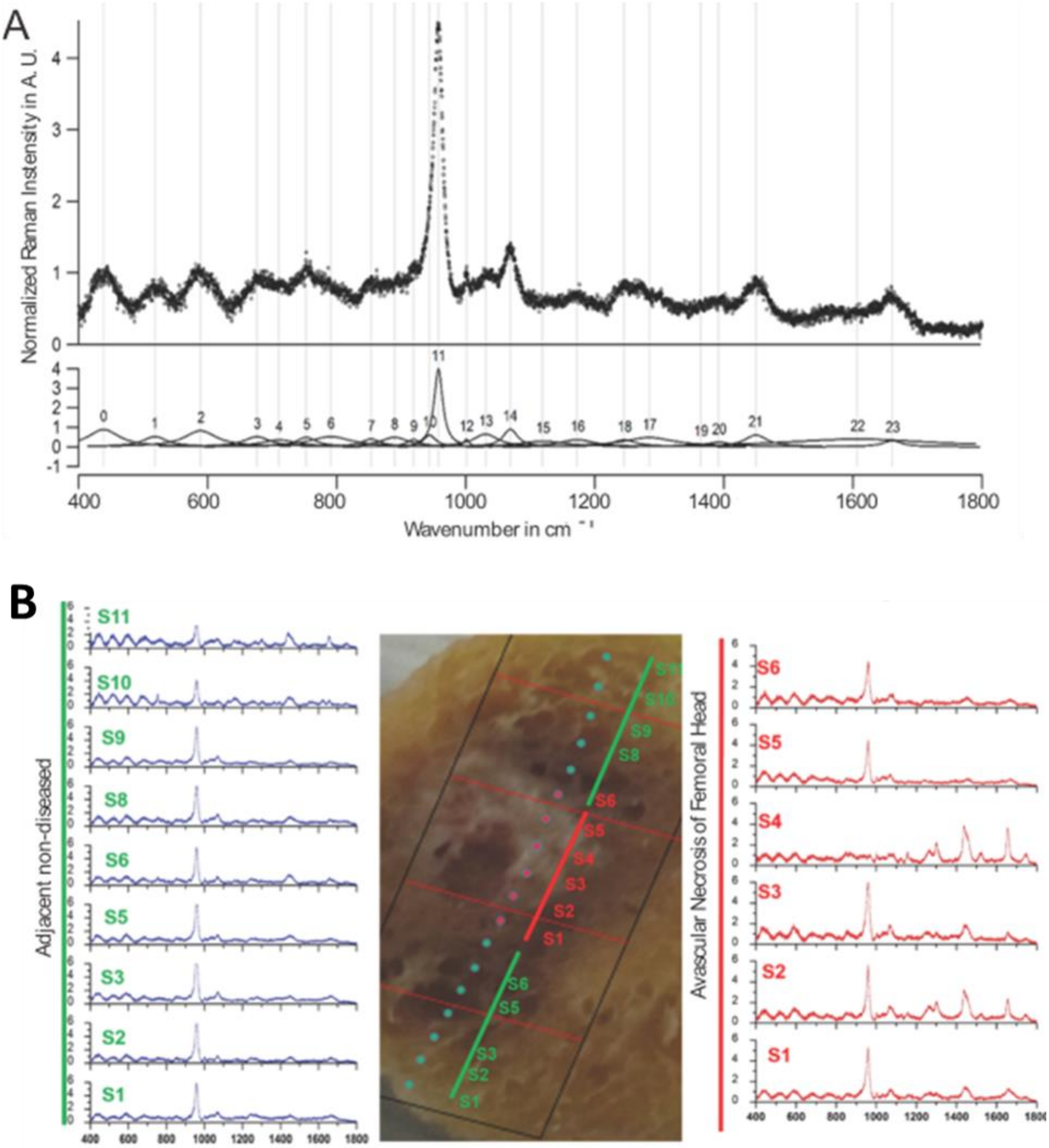

Supplementary Figure 5: Overview of Raman spectrum obtained in this study. A) Representative Raman spectrum for the femoral bone. X-axis represents the various peaks defined by Wavenumber and the Y-axis represents the intensity of the peaks in arbitrary units. Panel A describes the chemical identity of each of the Raman peaks in the spectrum. B) Representative image comparing Raman spectrum from line scan obtained from successive spots in the adjacent non-diseased and AVNFH region in a cross section of AVNFH trabecular bone. Panel marker regions Left and Right represent the consecutive line scans from the adjacent non-diseased and AVNFH regions respectively. Panel marked Middle represents the regions with in trabecular cross section that were marked as either adjacent non-diseased (Green spots S1-S6 and S8-S11) or as AVNFH (red, spots S1-S6) respectively.

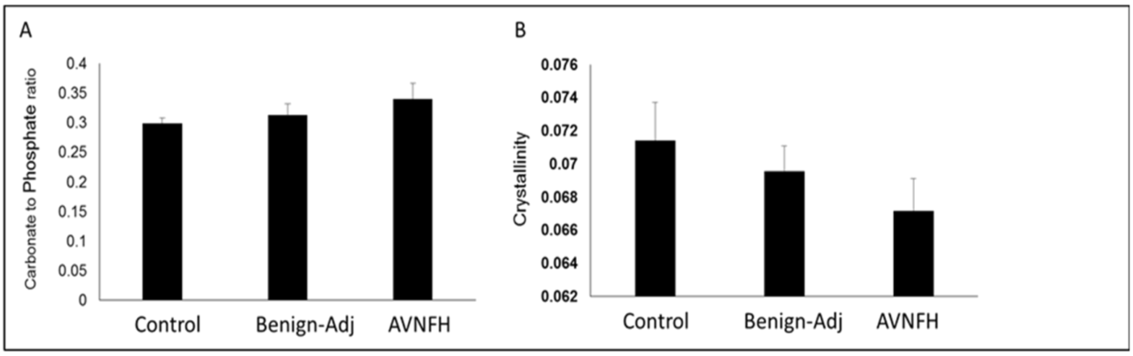

Supplementary Figure 6: A) Column plot of Micro-Raman spectroscopy data showing unchanged carbonate to phosphate substitution ratio and in control, adjacent benign and in AVNFH bone samples. B) Same as in A, but for Crystallinity. Crystallinity was reduced but not significant (P<0.08) in AVNFH when compared to controls

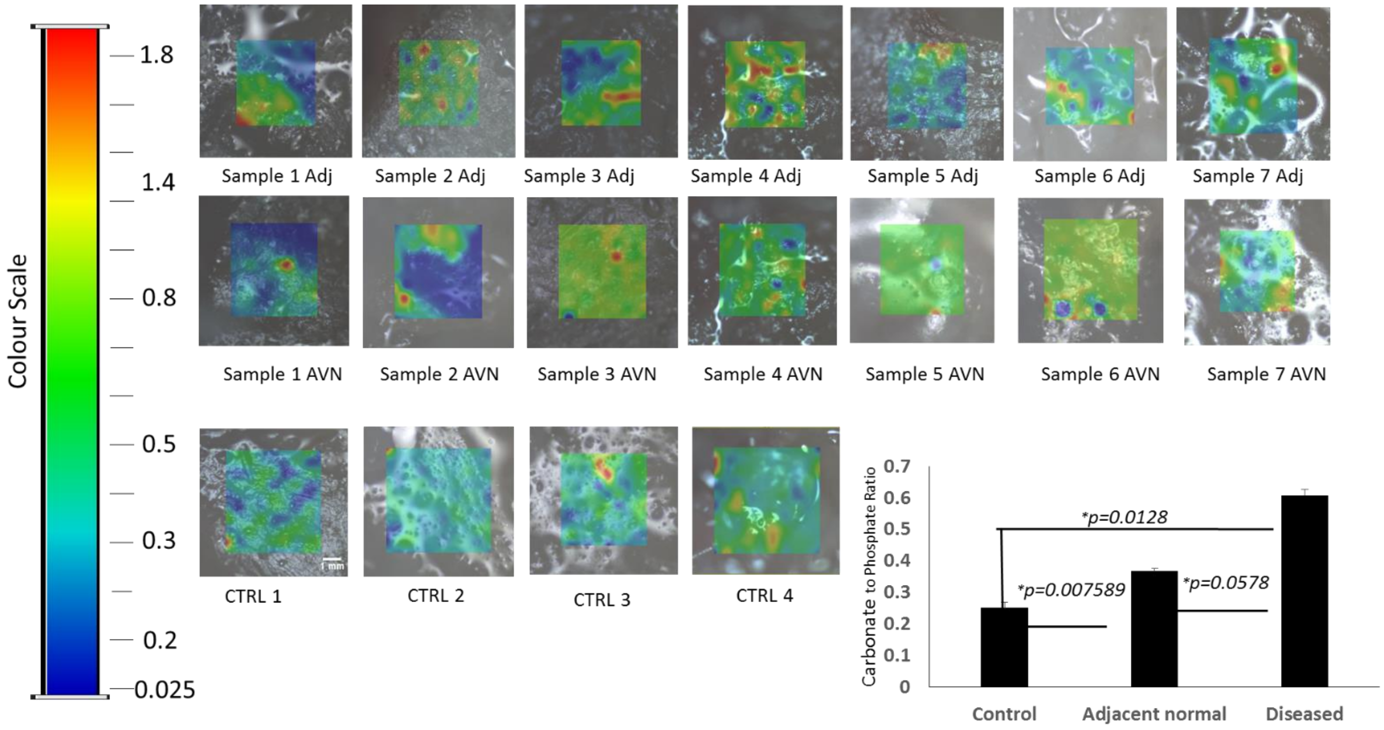

Supplementary Figure 7: Heat map showing the significant increase in carbonate to phosphate ratio displayed by diseased and Adjacent normal areas of AVNFH as compared to control bone samples. Colour scale from blue to red denotes the progressive increase in the carbonate to phosphate ratio with the least intensity depicted by blue colour and the maximum intensity shown by red colour. Quantification of the carbonate to phosphate ratio is shown as a bar plot with associated P values calculated using Student’s T-test.

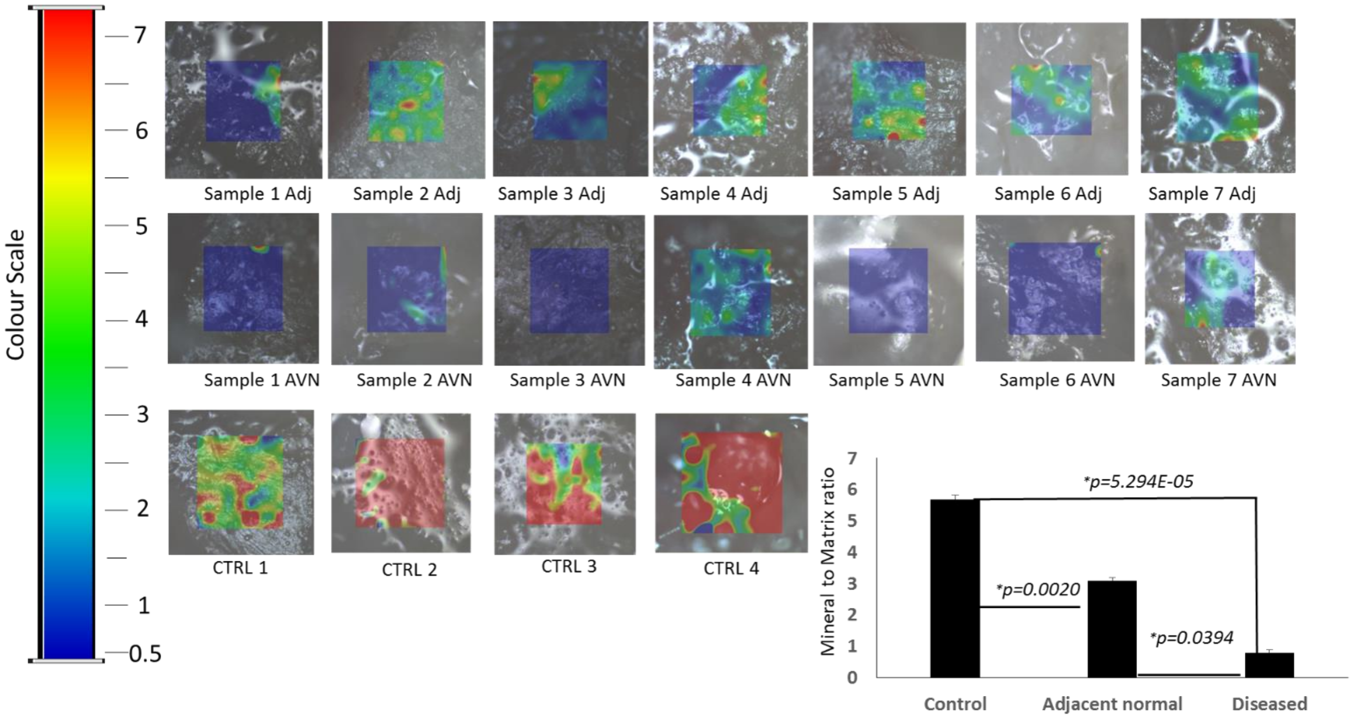

Supplementary Figure 8: Heat map showing the significant increase in Mineral to matrix ratio displayed by diseased and Adjacent normal areas of AVNFH as compared to control bone samples. Colour scale from blue to red denotes the progressive increase in the Mineral to matrix ratio with the least intensity depicted by blue colour and the maximum intensity shown by red colour. Quantification of mineral to matrix ratio is shown as a bar plot with associated P values calculated using Student’s T-test.

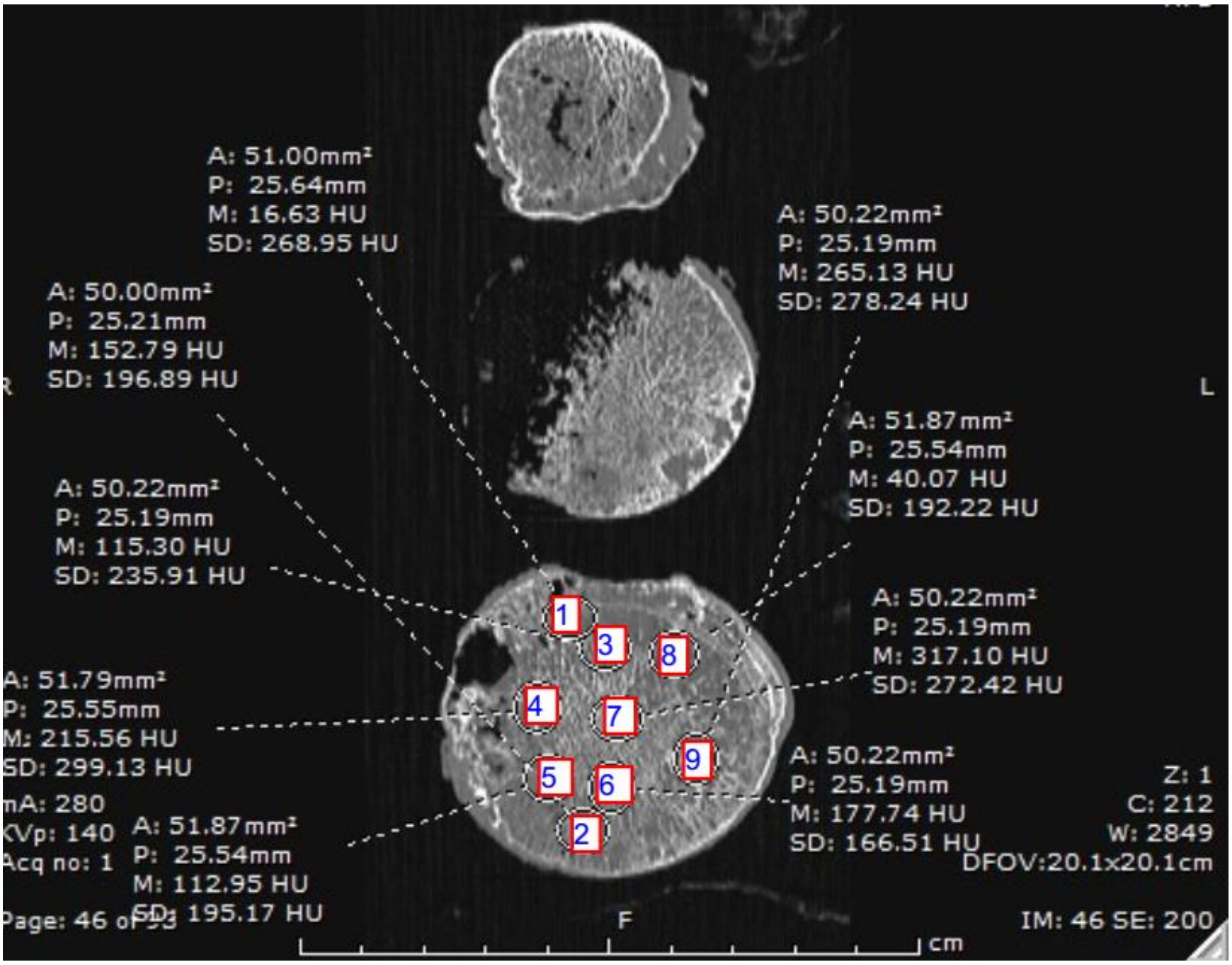

Supplementary Figure 9: Representative figure showing the region of interests (ROIs) in the Computer Assisted Tomography (CT) scans of AVNFH 1 bone sections selected for calculation of standardized CT measurements, represented by Hounsfield Units (HU, see main text for description). All the selected ROI's used for calculation of mean CT value (M) had an area (A) in the range of 49.61-51.87 mm^2 and Perimeter (P) in the range of 25.01-25.70 mm. On an average, 4 bone slices were used for the analysis. For each bone slice, an average of 5-9 ROI's were used for the calculation of the mean/median Hounsfield Units. For each patient, both average (M) and median values of CT intensity along with their corresponding standard deviation was calculated (see Figure 5 F).

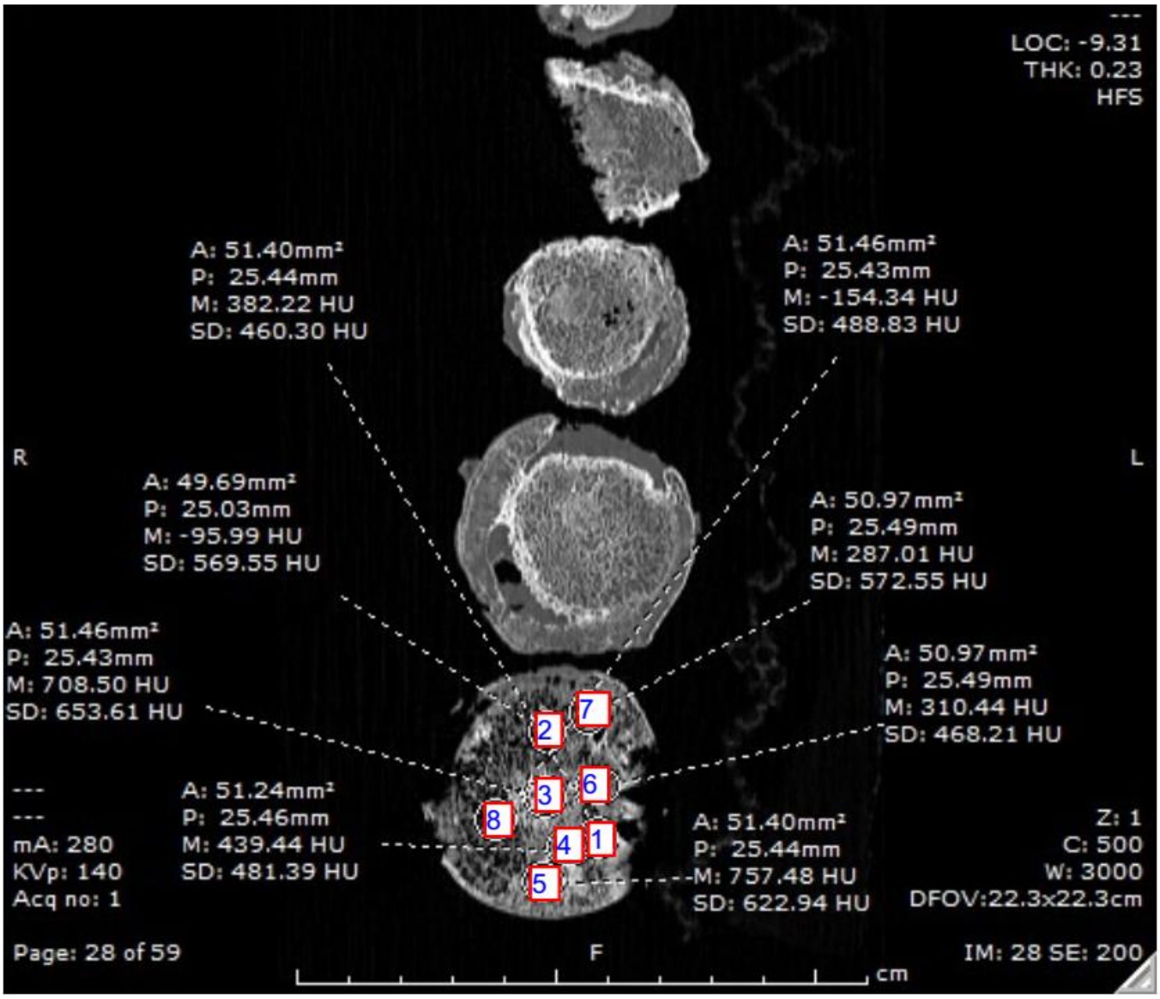

Supplementary Figure 10: Representative figure showing the region of interests (ROIs) in the Computer Assisted Tomography (CT) scans of AVNFH 2 bone sections selected for calculation of standardized CT measurements, represented by Hounsfield Units (HU, see main text for description). All the selected ROI's used for calculation of mean CT value (M) had an area (A) in the range of 49.36-51.87 mm^2 and Perimeter (P) in the range of 25.01-25.70 mm. On an average, 4 bone slices were used for the analysis. For each bone slice, average of 4-8 ROI's were used for the calculation of the mean/median Hounsfield value. For each patient, both average (M) and median values of CT intensity along with their corresponding standard deviation was calculated (see Figure 5 F).

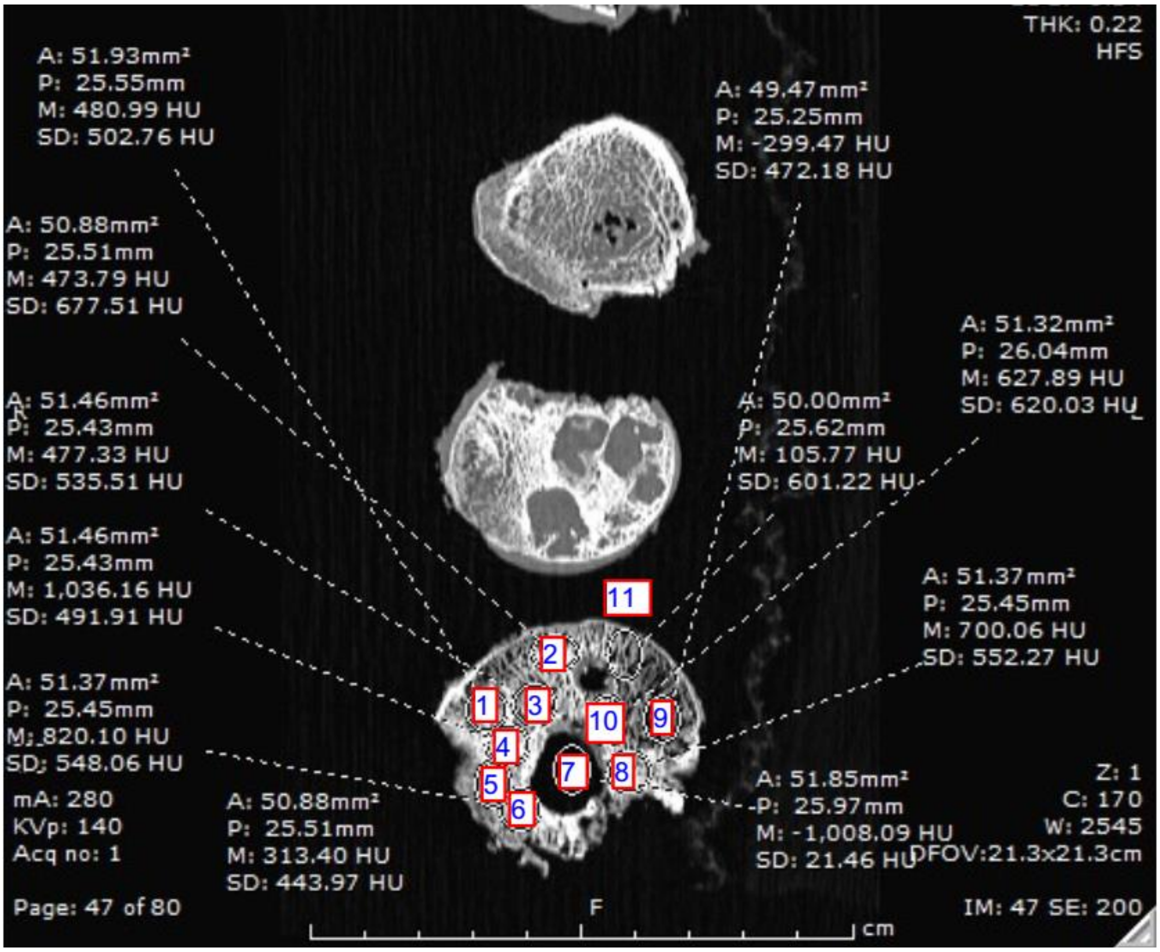

Supplementary Figure 11: Representative figure showing the region of interests (ROIs) in the Computer Assisted Tomography (CT) scans of AVNFH 3 bone sections selected for calculation of standardized CT measurements, represented by Hounsfield Units (HU, see main text for description). All the selected ROI's used for calculation of mean CT value (M) had an area (A) in the range of 49.36-51.87 mm^2 and Perimeter (P) in the range of 25.01-25.70 mm. On an average, 4 bone slices were used for the analysis. For each bone slice, an average of 2-11 ROI's were used for the calculation of mean/median Hounsfield value. For each patient, both average (M) and median values of CT intensity along with their corresponding standard deviation was calculated (see Figure 5 F).

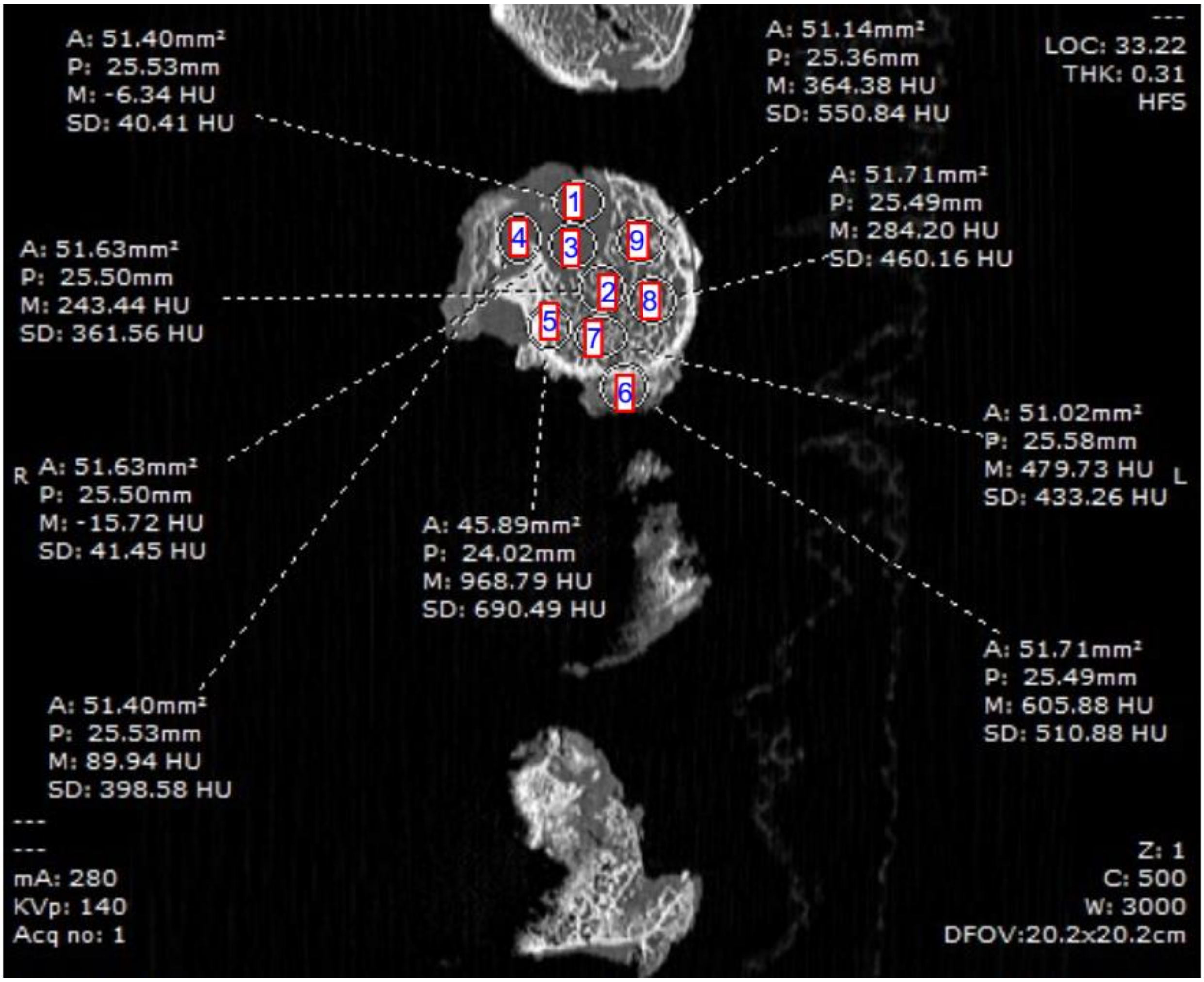

Supplementary Figure 12: Representative figure showing the region of interests (ROIs) in the Computer Assisted Tomography (CT) scans of AVNFH 4A bone sections selected for calculation of standardized CT measurements, represented by Hounsfield Units (HU, see main text for description). All the selected ROI's used for calculation of mean CT value (M) had an area (A) in the range of 49.36-51.87 mm^2 and Perimeter (P) in the range of 25.01-25.70 mm. On an average, 4 bone slices were used for the analysis. For each bone slice, an average of 2-11 ROI's were used for the calculation of mean/median Hounsfield Units. For each patient sample both average (M) and median values of CT intensity along with their corresponding standard deviation was calculated (see Figure 5 F).

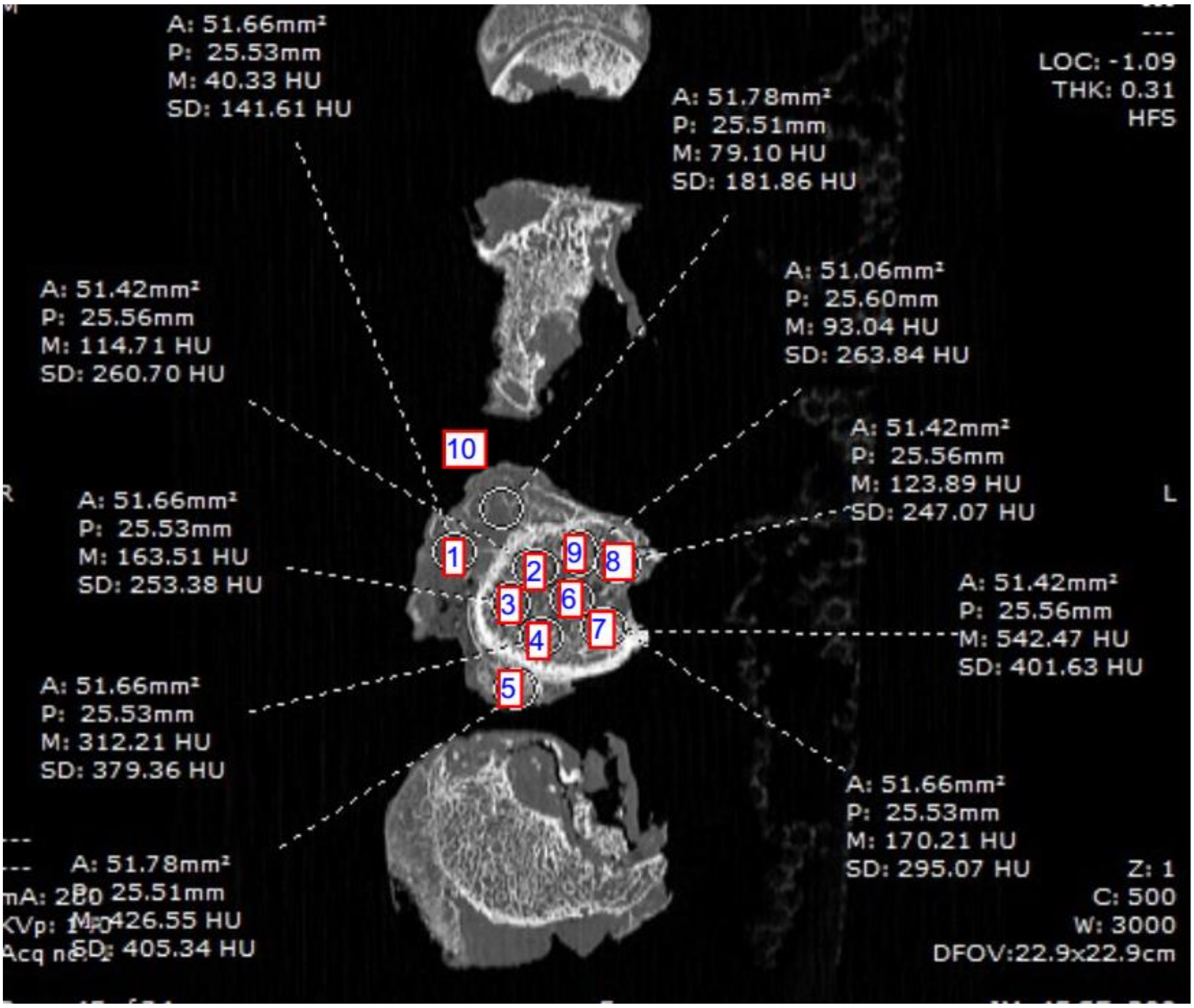

Supplementary Figure 13: Representative figure showing the region of interests (ROIs) in the Computer Assisted Tomography (CT) scans of AVNFH 4B bone sections selected for calculation of standardized CT measurements, represented by Hounsfield Units (HU, see main text for description). All the selected ROI's used for calculation of mean CT value (M) had an area (A) in the range of 49.36-51.87 mm^2 and Perimeter (P) in the range of 25.01-25.70 mm. On an average, 4 bone slices were used for the analysis. For each bone slice, an average of 2-11 ROI's were used for the calculation of mean/median Hounsfield Units. For each patient sample both average (M) and median values of CT intensity along with their corresponding standard deviation was calculated (see Figure 5 F).

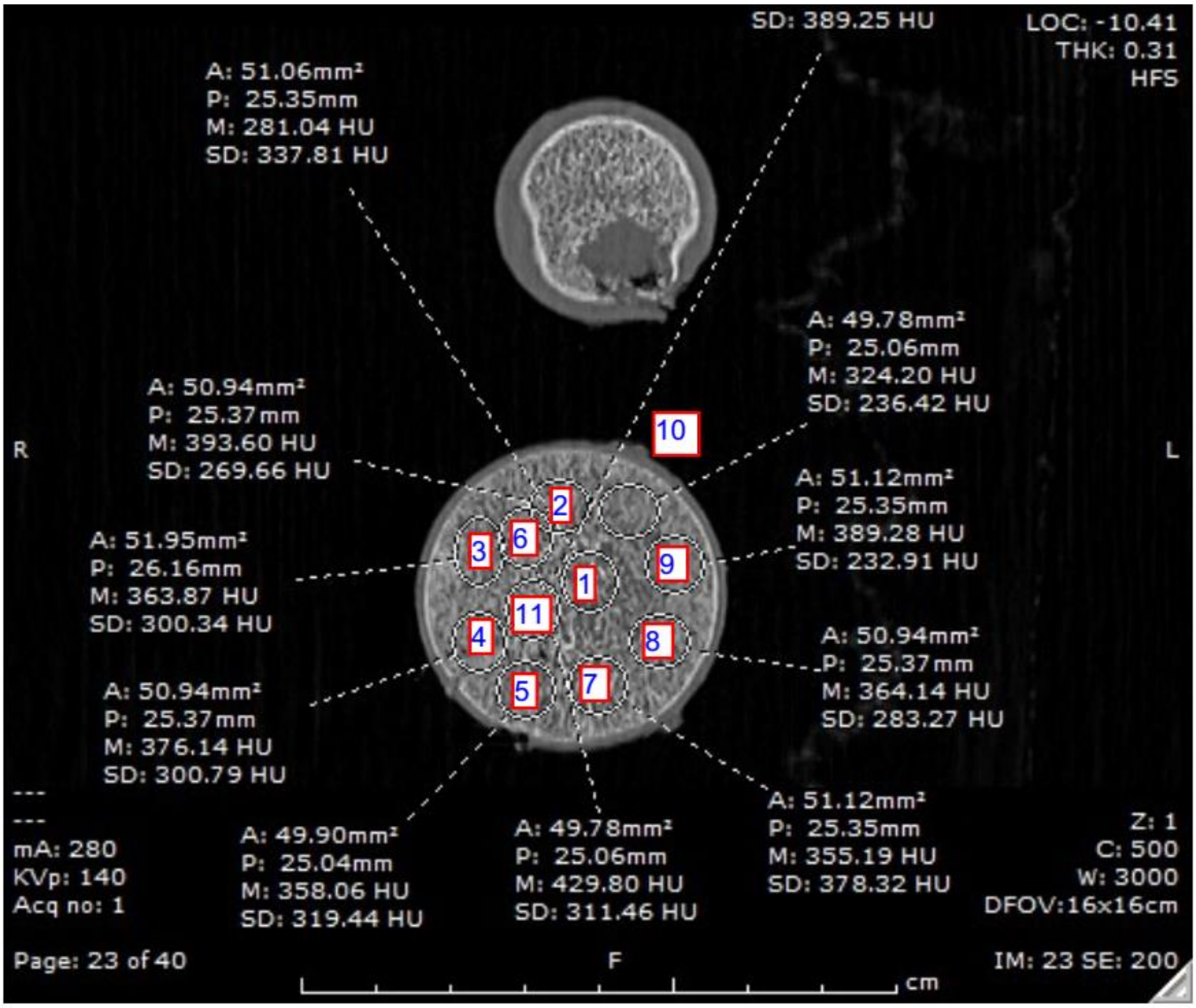

Supplementary Figure 14: Representative figure showing the region of interests (ROIs) in the Computer Assisted Tomography (CT) scans of AVNFH 5 bone sections selected for calculation of standardized CT measurements, represented by Hounsfield Units (HU, see main text for description). All the selected ROI's used for calculation of mean CT value (M) had an area (A) in the range of 49.36-51.87 mm^2 and Perimeter (P) in the range of 25.01-25.70 mm. On an average, 4 bone slices were used for the analysis. For each bone slice, an average of 2-11 ROI's were used for the calculation of mean/median Hounsfield units. For each patient sample both average (M) and median values of CT intensity along with their corresponding standard deviation was calculated (see Figure 5 F).

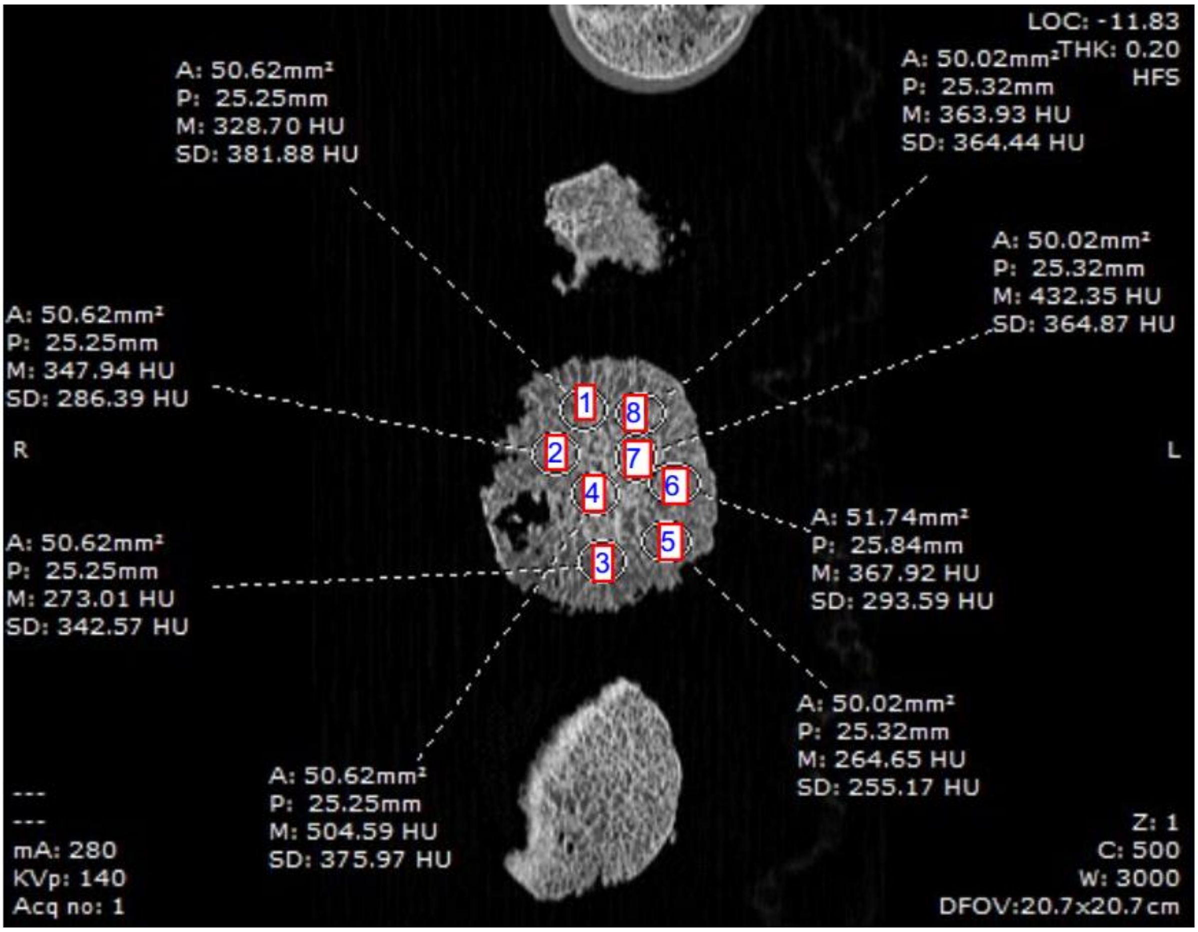

Supplementary Figure 15: Representative figure showing the region of interests (ROIs) in the Computer Assisted Tomography (CT) scans of AVNFH 6 bone sections selected for calculation of standardized CT measurements, represented by Hounsfield Units (HU, see main text for description). All the selected ROI's used for calculation of mean CT value (M) had an area (A) in the range of 49.36-51.87 mm^2 and Perimeter (P) in the range of 25.01-25.70 mm. On an average, 4 bone slices were used for the analysis. For each bone slice, an average of 2-11 ROI's were used for the calculation of mean/median Hounsfield Units. For each patient sample both average (M) and median values of CT intensity along with their corresponding standard deviation was calculated (see Figure 5 F).

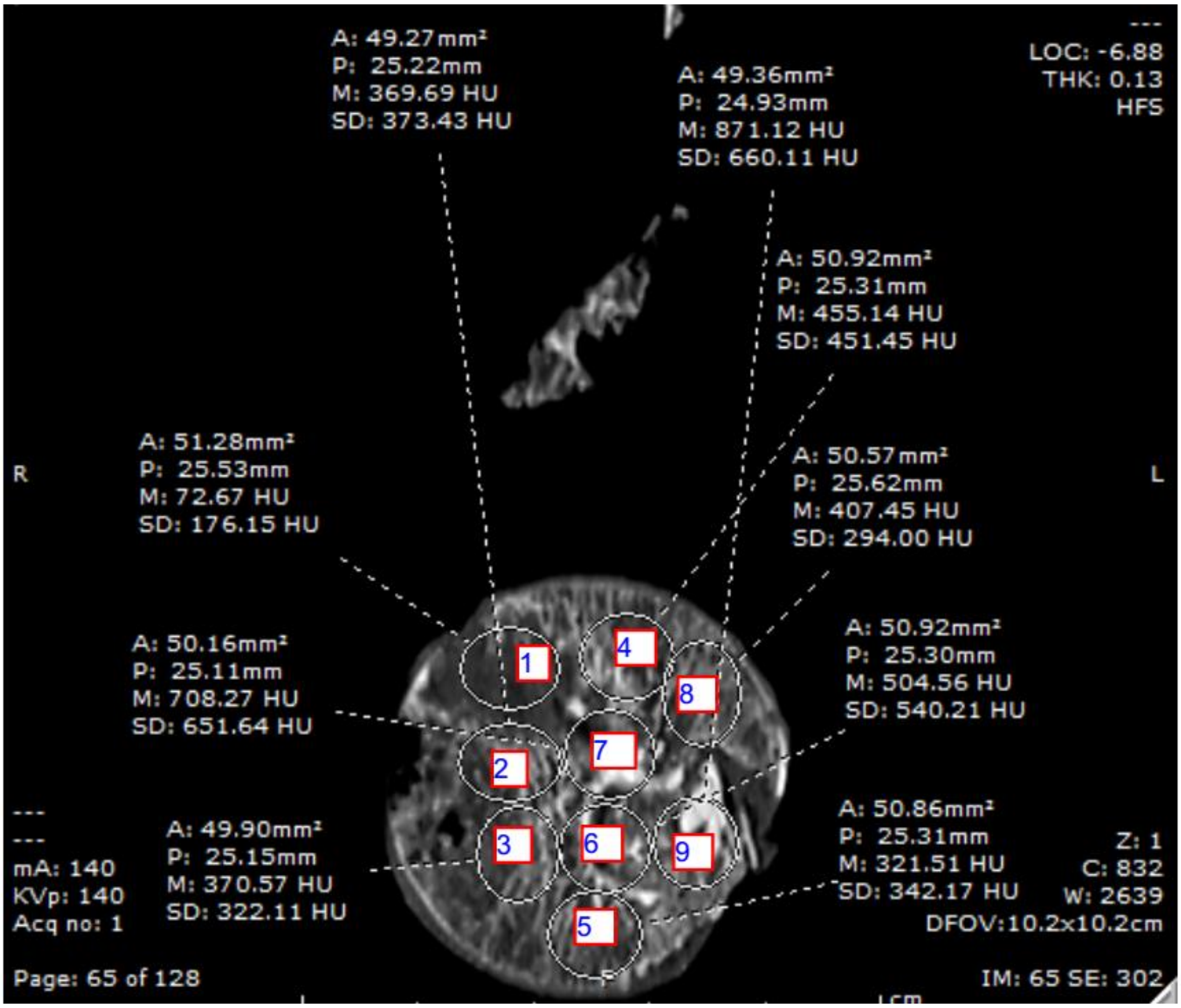

Supplementary Figure 16: Representative figure showing the region of interests (ROIs) in the Computer Assisted Tomography (CT) scans of Control 1 bone sections selected for calculation of standardized CT measurements, represented by Hounsfield Units (HU, see main text for description). All the selected ROI's used for calculation of mean CT value (M) had an area (A) in the range of 49.36-51.87 mm^2 and Perimeter (P) in the range of 25.01-25.70 mm. On an average, 4 bone slices were used for the analysis. For each bone slice, an average of 2-11 ROI's were used for the calculation of mean/median Hounsfield units. For each control sample both average (M) and median values of CT intensity along with their corresponding standard deviation was calculated (see Figure 5 F).

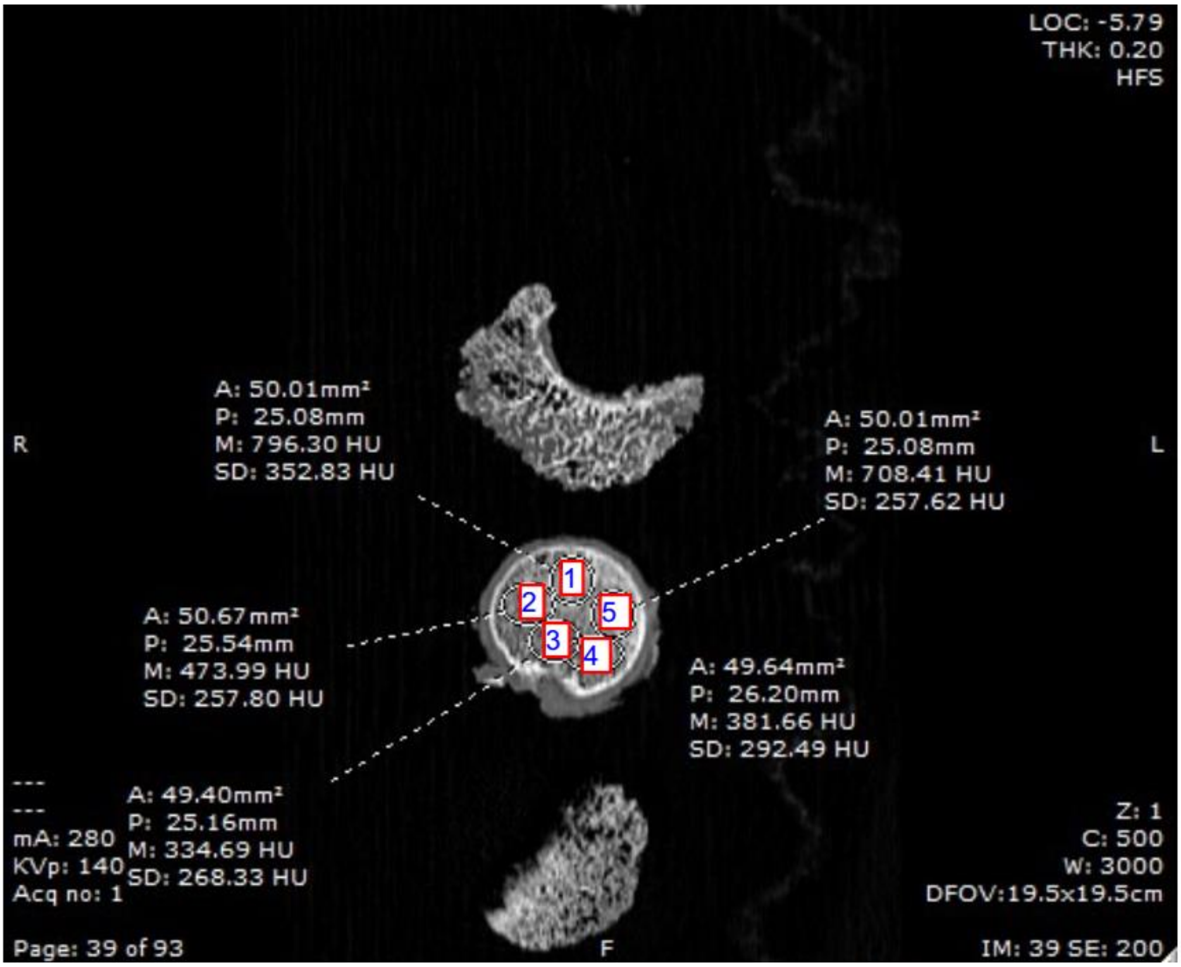

Supplementary Figure 17: Representative figure showing the region of interests (ROIs) in the Computer Assisted Tomography (CT) scans of Control 2 bone sections selected for calculation of standardized CT measurements, represented by Hounsfield Units (HU, see main text for description). All the selected ROI's used for calculation of mean CT value (M) had an area (A) in the range of 49.36-51.87 mm^2 and Perimeter (P) in the range of 25.01-25.70 mm. On an average, 4 bone slices were used for the analysis. For each bone slice, an average of 2-11 ROI's were used for the calculation of the mean/median Hounsfield Units. For each control sample both average (M) and median values of CT intensity along with their corresponding standard deviation was calculated (see Figure 5 F).

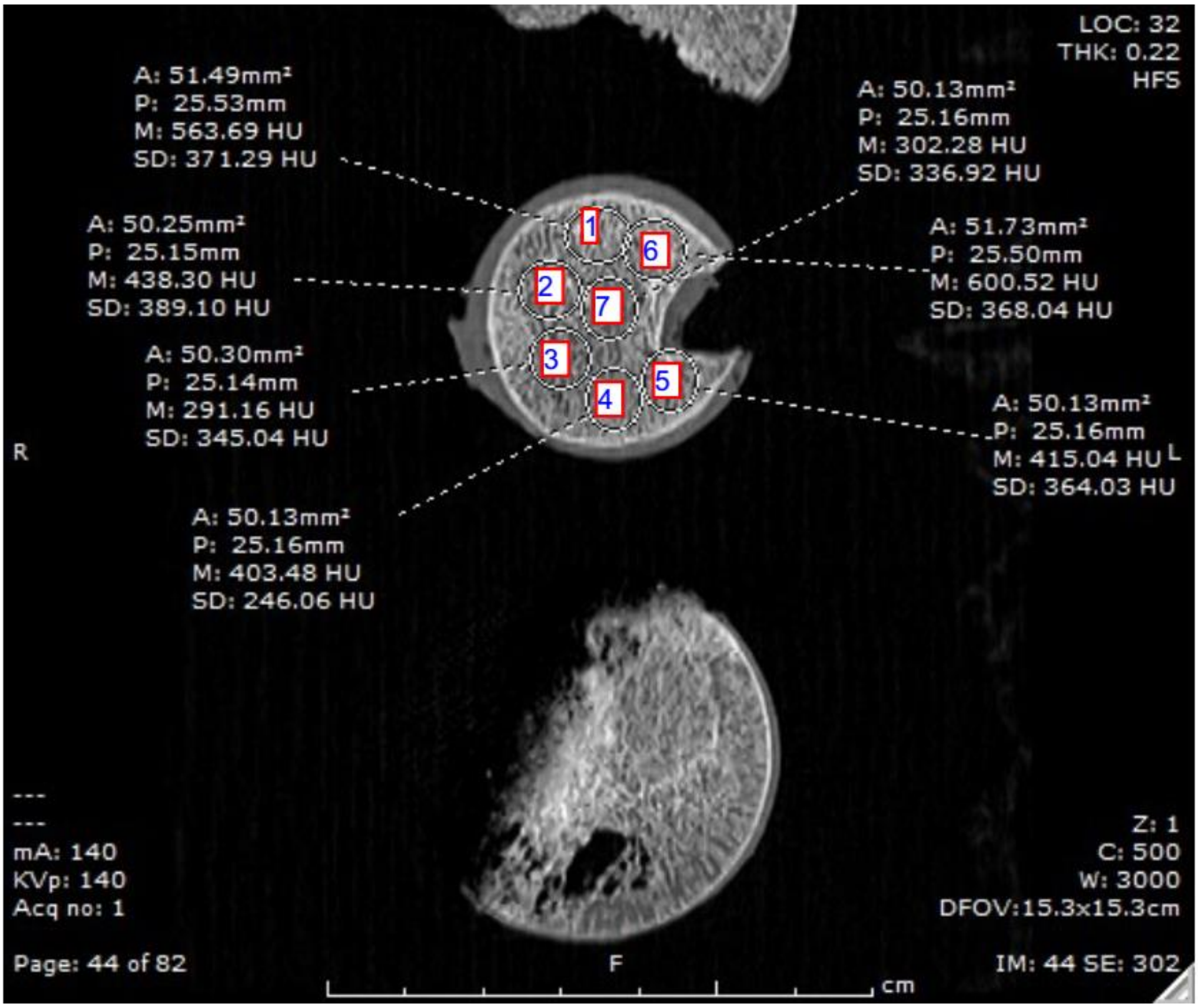

Supplementary Figure 18: Representative figure showing the region of interests (ROIs) in the Computer Assisted Tomography (CT) scans of Control 3 bone sections selected for calculation of standardized CT measurements, represented by Hounsfield Units (HU, see main text for description). All the selected ROI's used for calculation of mean CT value (M) had an area (A) in the range of 49.36-51.87 mm^2 and Perimeter (P) in the range of 25.01-25.70 mm. On an average, 4 bone slices were used for the analysis. For each bone slice, an average of 2-11 ROI's were used for the calculation of mean/median Hounsfield Units. For each control sample both average (M) and median values of CT intensity along with their corresponding standard deviation was calculated (see Figure 5 F).

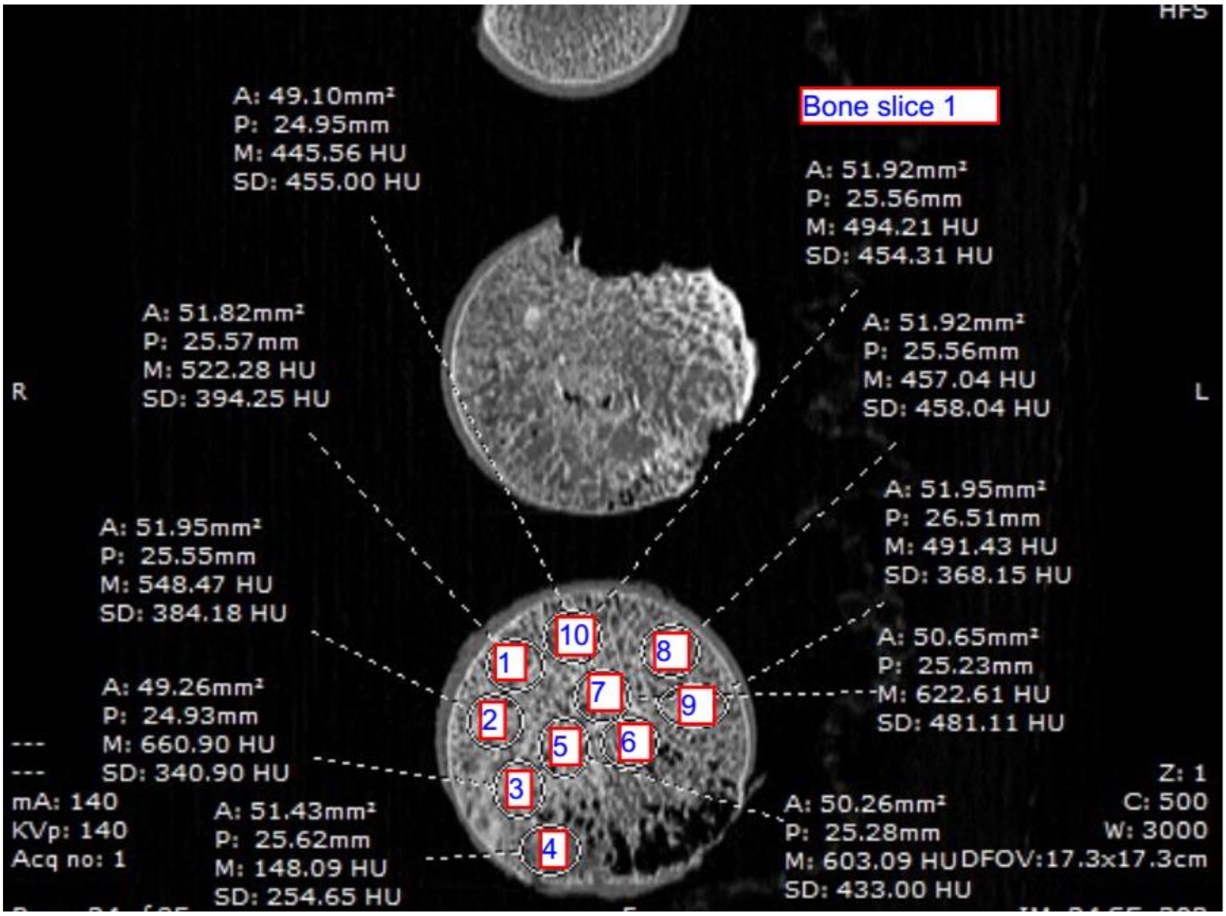

Supplementary Figure 19: Representative figure showing the region of interests (ROIs) in the Computer Assisted Tomography (CT) scans of Control 4 bone sections selected for calculation of standardized CT measurements, represented by Hounsfield Units (HU, see main text for description). All the selected ROI's used for calculation of mean CT value (M) had an area (A) in the range of 49.36-51.87 mm^2 and Perimeter (P) in the range of 25.01-25.70 mm. On an average, 4 bone slices were used for the analysis. For each bone slice, an average of 2-11 ROI's were used for the calculation of mean/median Hounsfield Units. For each control sample both average (M) and median values of CT intensity along with their corresponding standard deviation was calculated (see Figure 5 F).

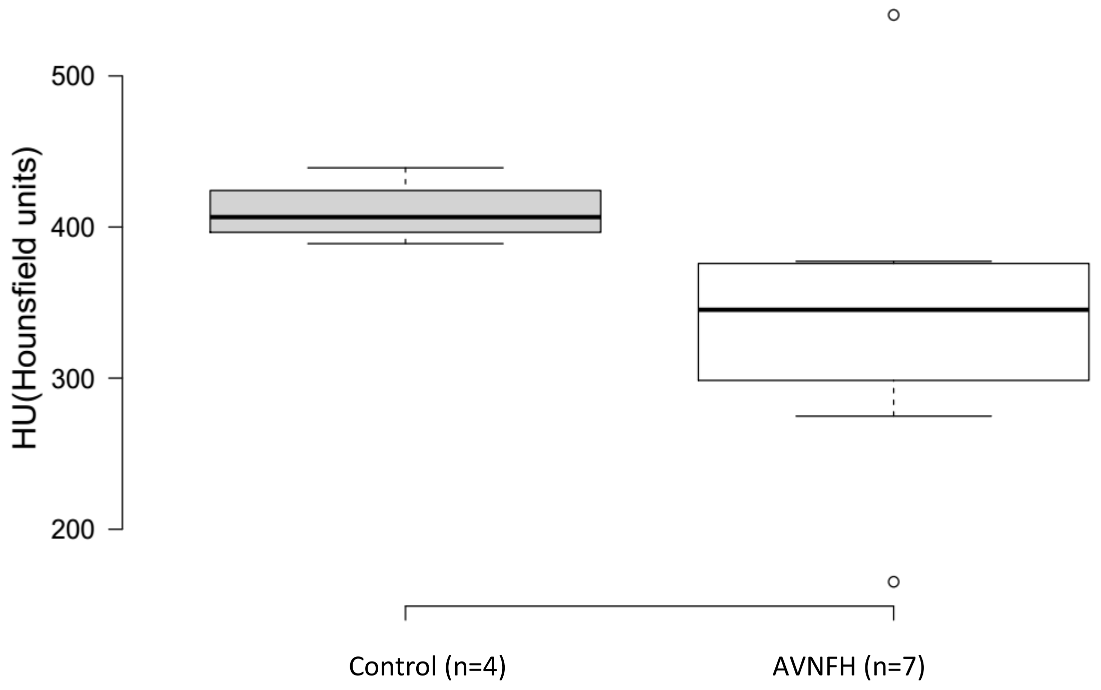

Supplementary Figure 20: Box plot showing the comparison of median CT intensities quantified using Hounsfield unit values (HU) for control (n=4) vs AVNFH (n=7) bone samples (see main text for details). Median HU values for AVNFH group was lower (not significant, P=0.245*)* when compared to the control group. Notably, 1/6 AVNFH specimen was an outlier with high HU values comparable to the control, resulting in the overall values being lower in AVNFH group (but not significant) compared to controls. This outlier effect could potentially be due to the presence of subchondral sclerotic areas in the AVNFH sample.

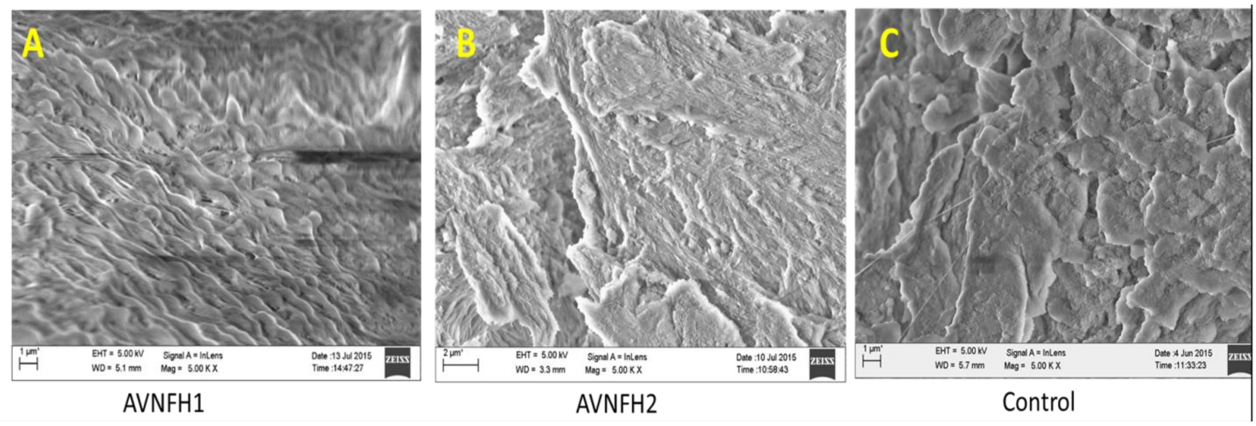

Supplementary Figure 21: Representative images of AVNFH and control bones taken using Scanning Electron Microscope (SEM) at a magnification of 5000x reveal irregular and disaggregated arrangement of collagen bundles and lamellar plates. A) Idiopathic B) Alcohol-induced AVNFH and C) Control bone from an individual having osteoporosis.

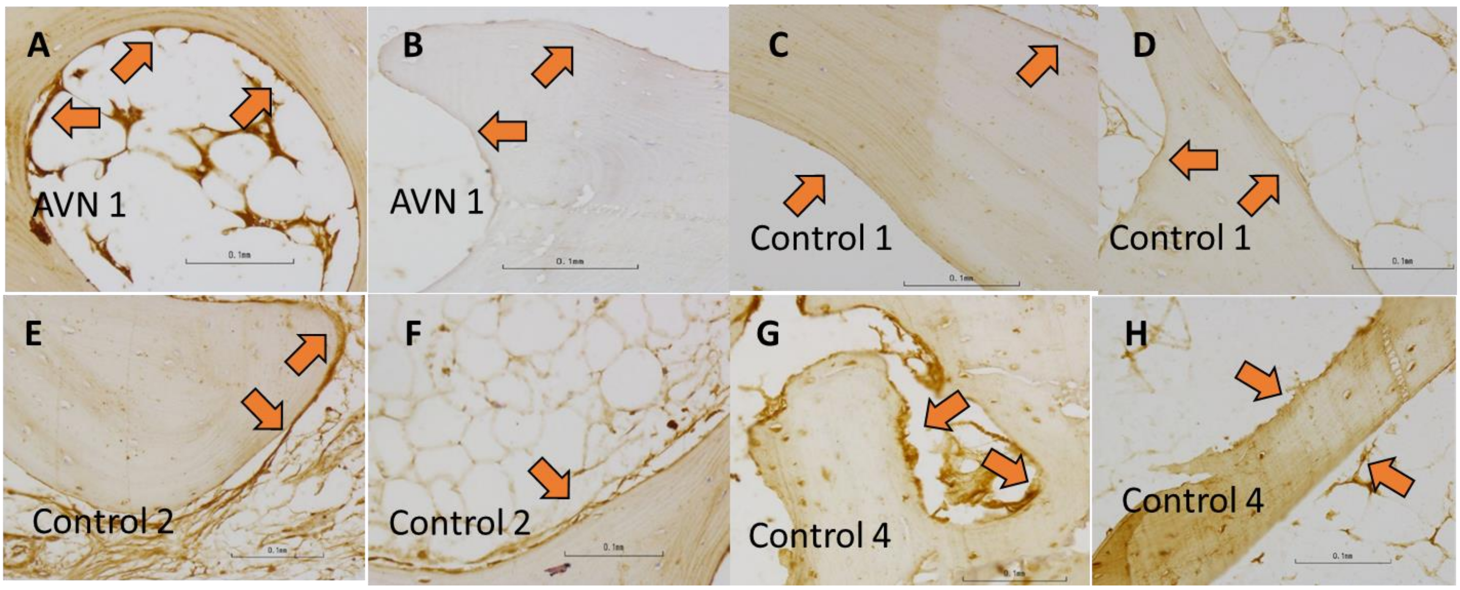

Supplementary Figure 22: Photomicrographs comparing osteocalcin staining in AVNFH (panels A and B) and control (fracture bone, panels C-H) bone samples in regions that are either proximal or distal to the site of necrosis (in case of AVNFH) or fracture callus (control). Osteocalcin staining in osteoblasts lining the bone are indicated in arrows. In AVNFH samples, strong osteocalcin staining was observed in osteoblasts present in the vicinity of necrotic regions (panel A) and weak to negligible staining was seen in osteoblasts lining the bone in area distal from the site of necrosis (panel B). In contrast, in majority of the controls, (panels C and D), weak to negligible osteocalcin staining was observed in the osteoblasts lining unaffected regions. In controls 2 and 4 (panels E, G), strong staining was seen in the osteoblasts in an area that has undergone fracture and repair. In contrast, in the same bone samples, weak to moderate osteocalcin staining was observed in unaffected regions (panels F and H). Overall, strong osteocalcin staining was observed in osteoblasts lining areas of active bone repair. In all cases, non-specific staining was observed in the marrow. All the images were photographed at 200x (refer to scale in the inset).

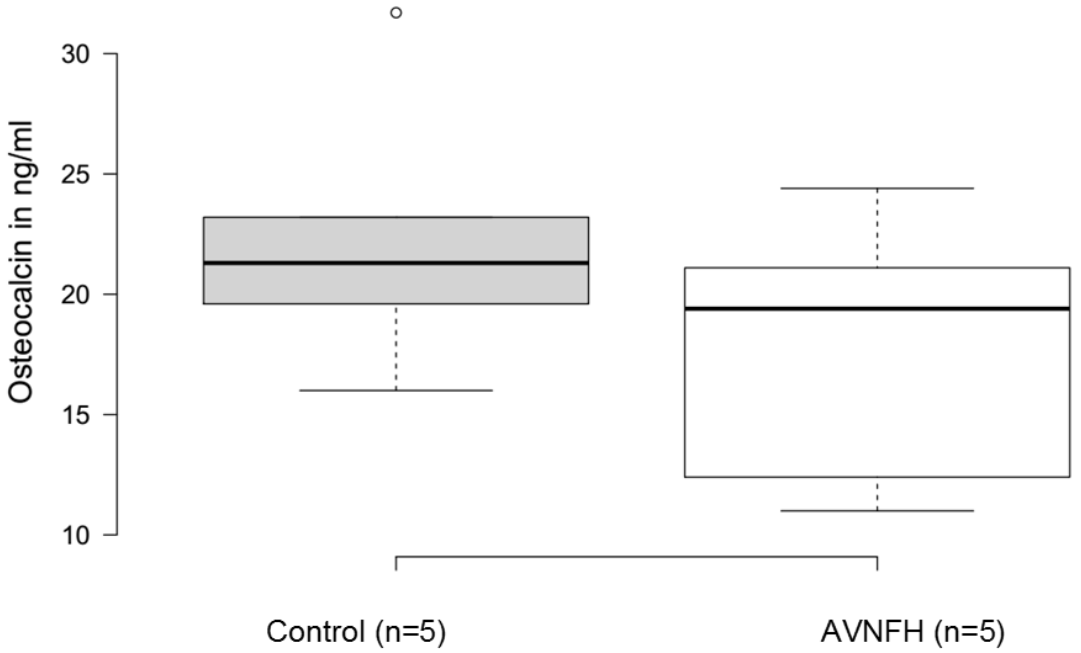

Supplementary Figure 23: Box plot showing the absolute levels (ng/ml) of osteocalcin in control (n=5) and AVNFH (n=5) serum. Overall there was no difference in the serum levels of osteocalcin between AVNFH patients and control individuals.

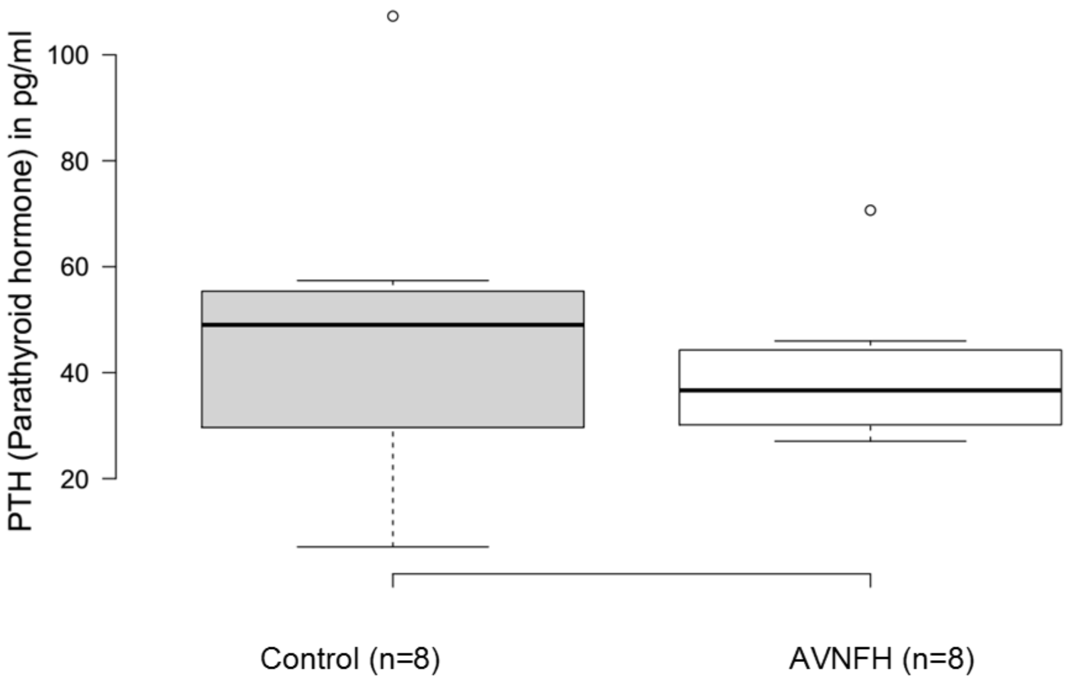

Supplementary Figure 24: Box plot showing the absolute levels (pg/ml) of parathyroid hormone (PTH) in control (n=8) and AVNFH (n=8) serum. Overall there was no difference in the serum levels of PTH between AVNFH patients and control individuals.

Compound Name	Precursor Ion (m/z)	Product Ion (m/z)	Dwell Time (ms)	Collision Energy (V)	Polarity
**Cystathionine**	**223.08**	**134.1/88.1**	**50**	**8**	**Positive**
**L- Tryptophan**	**207.1**	**147.1**	**50**	**10**	**Positive**
**S-(5’-Adenosyl)-L-methionine (SAM)**	**399.2**	**250.1/136.1**	**50**	**8**	**Positive**
**S-(5’-Adenosyl)-L-Homocysteine (SAH)**	**385.1**	**136.0**	**50**	**13**	**Positive**
**Arginine**	**175.12**	**70.1/60.2**	**50**	**17**	**Positive**
**Proline**	**116.07**	**70/43**	**50**	**33/17**	**Positive**
**Betaine**	**118.09**	**58.1**	**50**	**33**	**Positive**
**Ornithine**	**133.1**	**116/70.1**	**50**	**17**	**Positive**
**Methionine**	**150.06**	**56.1/61.1**	**50**	**21**	**Positive**
**Adenosine**	**268.1**	**137.1**	**50**	**12**	**Positive**
**Adenine**	**136.1**	**92**	**50**	**10**	**Positive**
**Homocysteine**	**135.2**	**34**	**50**	**15**	**Positive**
**Putrescene**	**89.1**	**55/72**	**50**	**10**	**Positive**
**Spermidine**	**146**	**112.1/72.1**	**50**	**18**	**Positive**
**Spermine**	**203**	**112.1/84.1**	**50**	**20**	**Positive**
**Vitamin B_6**	**170.1**	**152**	**50**	**17**	**Positive**
**Vitamin B_{12}**	**678.3**	**147.1/359.2**	**50**	**30**	**Positive**

Supplementary Table S1: List of SRM transitions and assay parameters used to measure metabolites in plasma of AVNFH patients and control individuals.

Peak No	Raman shift	Assignment	Component
1	437	V_2 PO_4^{3-}	Apatite mineral
2	517	Not characterized	Not characterized
3	586	V_4 PO_4^{3-}	Apatite mineral
4	680	Not characterized	Not characterized
5	766	Not characterized	Not characterized
6	860	Vcc Hydroxyproline	Collagen
7	958	$V_1PO_4^{3-}$	Apatite mineral
8	1002	Phenyl alanine	Collagen
9	1031	V_3 PO_4^{3-}	Apatite mineral
10	1067	$V_2CO_3^{2-}$	Apatite mineral
11	1168	C-O,C-C oxygen bridge	Hyaluronic acid
12	1255	=CH deformation	lipid
13	1300	CH_2 deformation	lipid
14	1375	COO-symmetric stretch, CH_3 deformation	Glycosoaminoglycans
15	1446	CH_2 wag	Collagen
16	1571	Not characterized	Not characterized
17	1658	Amide 1	Collagen
18	1746	V C=0	Lipid

Supplementary Table S2: Reference annotation and functional moiety assignment for Raman spectral peaks in a control bone sample.

Mass spectrometry data set	Samples used for correlation analysis	Pearson Correlation : homocysteine versus vitamin B_{12} and P value	Pearson Correlation : homocysteine versus vitamin B_6 and P value	Pearson Correlation : homocysteine versus spermine and P value	Pearson Correlation : homocysteine versus spermidine and P value
1	AVNFH (n=14) and Controls (n=14)	-.45572, 0.01431	-.44526, 0.015568	0.3854, 0.0389	0.3999, 0.0316
2	AVNFH (n=11) and Controls (n=12)	-.79626, <0.00001	-.80247, <0.00001	0.6259, 0.0014	0.6616, 0.0005
3	AVNFH (n=5) and Controls (n=5)	-.59635, 0.014832	-.48348, 0.05	0.5731, 0.0203	0.5388, 0.0312

Supplementary Table S3: Summary of Pearson correlation between homocysteine and vitamins B_{12}, B_6 as well as spermine and spermidine, in plasma samples from AVNFH and control individuals. Mass spectrometry analysis was conducted in three independent groups and the raw spectral data was used for the correlation analysis as described in the main text.

AVNFH Bone (n= 13)		Control Bone (n=7)
Age (Median, Range)	51(35-56)	65 (54-79)
Male	9	5
Female	4	2
Stage 1		
Stage 2b	1	
Stage 3	2	
Stage 4	10	
**AVNFH Plasma (n= 30)**		**Control Plasma (n=31)**
Age (Median, Range)	48 (23-65)	48 (23-65)
Male	23	26
Female	7	5
Idiopathic	4	
Alcoholic/smoking	8	
Post-Traumatic	9	
Steroid	1	
Pregnancy	2	
Hyperurecimia plus pregnancy	1	
Sickle cell anemia	4	
Ankylosing spondylitis	1	
Stage 1		
Stage 2b	2	
Stage 3	4	
Stage 4	24	

Supplementary Table S4: Summary of clinical data associated with prospectively collected AVNFH and control blood samples.

Clinical Parameter	AVNFH (n=69)	Control (71)
Age Median	41	42
Age Range	23-68	24-70
**Gender**		
Male	54	56
Female	15	15
**Classification**		
Unilateral AVNFH	21	
Bilateral AVNFH	48	
**Etiology**		
Idiopathic	24	
Post-traumatic	12	
Alcohol/Smoking	11	
Steroid	12	
Alcohol and steroid	1	
Sickle cell Anemia	3	
Sickle cell anemia and pregnancy	1	
Infection	2	
Ankylosing spondylitis	1	
Ankylosing spondylitis and albuminuria	1	
Pregnancy	1	
MRI	20	

Supplementary Table S5: Clinical characteristics of AVNFH patients and control individuals whose clinical chemistry data was used for retrospective analysis.

Clinical Parameter	P-value T test	P value glm test	Co-efficients
Random Glucose*	0.006472	0.03156	-0.021840
Hemoglobin*	0.000138	1.38×10^{-5}	-0.555503
Serum Creatinine*	0.010307	2.5×10^{-4}	6.508596

Supplementary Table S6: Results of multivariate analysis carried out using a logistic regression model with generalized linear model function in the Stats package R. Random glucose, Haemoglobin and Serum creatinine were found to be significantly altered parameters with p value < 0.05

S.No	Sample ID	Age	Gender	Age range	Reference values for Metropolis Diagnostic Labs in pg/ml	Observed Value of CTX measured in pg/ml
1	AVN1	46	Male	30-50	<584	2110*
2	AVN2	50	Male	30-50	<584	422.9
3	AVN3	38	Male	30-50	<584	311.4
4	AVN4	45	Male	30-50	<584	593.1*
5	AVN7	53	Male	50-70	<704	895*
6	AVN8	50	Male	30-50	<584	578
7	AVN9	42	Male	30-50	<584	319.7
8	Control1	48	Male	30-50	<584	769.1*
9	Control2	48	Male	30-50	<584	515
10	Control3	38	Male	30-50	<584	510
11	Control4	53	Male	50-70	<704	285.9
12	Control5	38	Male	30-50	<584	557.8
13	Control7	37	Male	30-50	<584	479

Supplementary Table S7A: Results of CTX which is a bone resorption test carried out in the blood plasma of AVN (n=7) patients and Control (n=6) individuals in the age group 37-53. The normal reference values from Metropolis Diagnostic labs where the CTX test was conducted for the age groups 30-50 years male is <584 and for 50-70 years male is <704. CTX values in 3/7 AVN patients (AVN1, AVN4 and AVN7, indicated by asterisk) tested were above the normal reference threshold and only 1/7 controls (Control 1, indicated by asterisk) had a value that exceeded this reference threshold.

S.No	Sample ID	Age	Gender	Observed Value of CTX measured in pg/ml	Reference range of CTX in Mayo clinic labs	Reference range of CTX in Quest Diagnostics	Reference range of CTX in Arup Lab
1	AVN5	24	Male	2110*	120-946	87-1200	87-1200
2	AVN6	29	Male	422.9	120-946	87-1200	87-1200
3	Control6	29	Male	311.4	120-946	87-1200	87-1200
4	Control8	28	Male	593.1	120-946	87-1200	87-1200
5	Control9	23	Male	895	120-946	87-1200	87-1200

Supplementary Table S7B: Results of CTX which is a bone resorption test carried out in the blood plasma of AVN (n=2) patients and Control (n=3) individuals in the age group 23-29. These tests were carried out in the Metropolis diagnostic laboratory that did not have an established reference range for CTX in patients below the age of 30 years. To address this caveat, reference ranges established by three independent diagnostic laboratories (Mayo clinic labs, Quest Diagnostic labs and Arup Lab), were used as reference for data reporting. Importantly, only 1/2 AVN cases (AVN5, indicated by asterisk) had CTX values beyond the reference threshold. None of the controls had CTX values beyond the reference threshold.

Supplementary Methods

Detailed methods used for Metabolomics analysis of plasma samples

Metabolomics

High-performance liquid chromatography (HPLC) grade acetonitrile, methanol and water were purchased from Fisher scientific (Mumbai, India). For extraction of metabolome, plasma samples were homogenized in 1:4 ice cold water: methanol mixture containing equimolar mixture of 2 standard compounds, Zeatine, $^{[15N]}$2-Tryptophan. This was followed by de-proteinization using 3 KDa molecular weight cut-off Amicon filters and drying the extract. Following this, the extract was re-suspended in injection solvent (water: acetonitrile, 95:5, containing 0.1 % formic acid) and analyzed by liquid chromatography- mass spectrometry (LC-MS/MS). The analysis was carried on a total sample size of AVNFH n=30 and Control n=31 patients in 2 different experiments, first on Cohort 1 (AVNFH n=14 and Control n=14) and Cohort 2 &3 (AVNFH n=16 and Control n=17). The extraction procedures for the samples belonging to cohorts 1-3 were the same as described above. The conditions used for HPLC and LC- MS/ MS analysis for the samples belonging to three cohorts are separately given as follows.

For Cohort 1 (AVNFH n=14 and Control n=14), the LC-MS/MS analysis was carried out on a Micromass Quattro Micro™, Waters Inc., Manchester, UK and Agilent 6420 triple quadrupole, Agilent Technologies, Santa Clara, CA coupled to Waters and Agilent HPLC system, respectively. The chromatographic separation was performed

on Micromass Quattro Micro™ by using Zorbax Eclipse XDB-C18 column (50 × 4.6 mm id.; 1.8 µm, from Agilent Technologies, Santa Clara, CA) wherein the column was maintained at a temperature of 37^{0}C. The mobile phase was composed of Solvent A (0.1% formic acid in water) and B (Acetonitrile) in gradient mode of elution. The binary pump flow rate for the separation was set at 0.2 mL/min with a gradient spanning 2% B to 95% B over a 25-minute time period. Metabolites were detected using Single Reaction Monitoring (SRM) using a Micromass Quattro Micro™ interfaced with an ESI source. Data acquisition was performed using MassLynx software. For mass spectrometry, the capillary voltage was maintained between 3.0–3.5 kV, and the cone voltage was kept at 20V unless otherwise stated. Nitrogen was used as de-solvation and nebulization gas. The source and de-solvation temperatures were set at 1000C. Mass spectrum for each metabolite was recorded using SRM, by selecting the parent ion in Q1 and specific fragment ions in Q3. Peak width at half height for Q1 and Q3 were set at 1 Da. Argon was used as collision gas, and the pressure in the collision cell was maintained at 3.5-4 mbar.

For Cohort 2 & 3 (AVNFH n=16 and Control n=17), 4 Nitro tyrosine was used as an internal standard keeping all the other extraction procedures identical to that of Cohort 1. Chromatographic separation was performed on Agilent system by using Kinetex C-8 column (4.6 x 250 mm; 5µm from Phenomenex, CA, USA). The mobile phase was composed of Solvent A (0.1% formic acid in water) and B (Acetonitrile) in gradient mode of elution. The elution started with 5 % solvent B, followed by linear gradient to 50% solvent B from 0 to 10 min. Following this, solvent B was increased from 50% to 95 % for up to 12 min. Equilibrium of solvent B was attained by 14 min and then returned to initial condition using a linear gradient from 14 to 15 min. The flow rate of mobile phase was kept at 0.6 mL/min and column temperature was maintained at 35

^{0}C. Mass spectrometer was operated in positive ion ESI mode, using the capillary voltage 3853 V, Gas flow of 11.0 L/min, Gas temperature at 300^{0}C and nebulizer pressure equal to 40.0 Psi.
